# IFITM proteins are key entry factors for porcine epidemic diarrhea coronavirus

**DOI:** 10.1128/jvi.02028-24

**Published:** 2025-05-12

**Authors:** Lilei Lv, Huaye Luo, Jingxuan Yi, Kang Zhang, Yanhua Li, Wu Tong, Yifeng Jiang, Yanjun Zhou, Guangzhi Tong, Changlong Liu

**Affiliations:** 1 Shanghai Veterinary Research Institute, Chinese Academy of Agricultural Sciences 118161 , Shanghai, China; 2 College of Animal Science and Technology, Guangxi University 622309 , Nanning, China; 3 College of Veterinary Medicine, Yangzhou University 614704, Yangzhou, China; 4 Jiangsu Co-Innovation Center for the Prevention and Control of Important Animal Infectious Disease and Zoonosis, Yangzhou University 38043, Yangzhou, China; Loyola University Chicago - Health Sciences Campus, Maywood, Illinois, USA

**Keywords:** IFITMs, PEDV, virus entry, coronavirus, porcine intestinal organoids

## Abstract

Porcine epidemic diarrhea virus (PEDV) is a highly contagious coronavirus that poses a substantial threat to the global swine industry. However, our current understanding of the host factors crucial for PEDV infection remains limited. To identify these host factors, we conducted a genome-wide CRISPR/Cas9 gene knockout screen using a PEDV-permissive cell line. Our results indicate that the endogenous expression of human interferon-inducible transmembrane protein 3 (IFITM3) enhances PEDV entry and replication. Silencing or eliminating endogenous IFITM3 in Huh7 cells significantly suppressed PEDV entry, whereas reintroducing IFITM3 partially restored susceptibility to PEDV. Overexpression of human IFITM3 or IFITM2, but not IFITM1, in Huh7.5 cells substantially increased PEDV entry and replication. Importantly, our results suggest that human IFITM3 influences PEDV entry at a later stage. Furthermore, the overexpression of porcine IFITM1 significantly enhanced PEDV infection in LLC-PK1 cells, whereas the overexpression of porcine IFITM2/3 did not produce similar effects. Notably, removing the C-terminal 15 amino acids of porcine IFITM2/3 resulted in increased PEDV entry. Coimmunoprecipitation analyses showed that all IFITMs interacted with the PEDV S1 protein, indicating a direct role in the viral entry process. Additionally, porcine IFITM1 colocalized with the PEDV S protein at the cell nuclear periphery and enhanced PEDV infection in porcine small intestinal organoids. Overall, our results suggest that IFITMs are critical in facilitating PEDV entry into cells. Targeting IFITMs may provide a promising strategy for controlling PEDV transmission and developing interventions to mitigate the virus’s impact on the swine industry.

**IMPORTANCE:**

Understanding the mechanisms underlying porcine epidemic diarrhea virus (PEDV) infection is vital for addressing its significant impact on the swine industry. This study reveals that interferon-inducible transmembrane (IFITM) proteins, particularly human IFITM3 and porcine IFITM1, play crucial roles in facilitating PEDV entry and replication. By elucidating these molecular interactions, the research highlights the potential of IFITMs as therapeutic targets for managing PEDV infections and paves the way for antiviral strategies. Moreover, this research extends beyond PEDV management, underscoring the critical role of host factors in controlling the spread of pathogenic coronaviruses.

## INTRODUCTION

Porcine epidemic diarrhea virus (PEDV) is a highly contagious coronavirus that causes severe enteric disease in pigs, leading to significant economic losses in the swine industry worldwide. PEDV was first identified in the 1970s in Europe and has spread globally, causing devastating outbreaks in Asia, North America, and Europe. The virus primarily affects young piglets, causing watery diarrhea, vomiting, dehydration, and high mortality rates that can reach 100% in suckling piglets (1). PEDV belongs to the genus *Alphacoronavirus* within the family Coronaviridae. The PEDV genome is a single-stranded, positive-sense RNA approximately 28 kb in length. Structurally, the PEDV genome consists of a 5´ cap structure followed by a polyadenylated tail at the 3´ end, which is typical of coronaviruses. Genome organization is characterized by seven open reading frames (ORFs): the 5´-proximal ORF1a and ORF1b, which encode nonstructural proteins involved in various aspects of viral replication, and the downstream ORFs, which encode structural proteins, including spike (S), envelope (E), membrane (M), and nucleocapsid (N) proteins, as well as several accessory proteins (2).

The S protein, a major structural component of PEDV, plays a crucial role in viral entry and pathogenesis (3). The S protein is a type I transmembrane glycoprotein that mediates attachment to host cells and facilitates membrane fusion, enabling the virus to infect susceptible cells. The S protein consists of three segments: a large ectodomain, a single-pass transmembrane anchor, and a short intracellular tail. In structural studies of other coronaviruses, the ectodomain consists of a receptor-binding subunit S1 and a membrane-fusion subunit S2. The S1 subunit is responsible for recognizing and binding to the host cell receptor. This interaction initiates the viral entry process, enabling the virus to attach to the host cell surface. The S2 subunit mediates membrane fusion, bringing the viral and cellular membranes together and ultimately enabling the viral genome to enter the host cell. The specific binding between the S1 protein and cell receptors is the critical first step for PEDV entry, which is essential for understanding the viral host range, tissue tropism, infection process, and potential cross-species transmissibility (4).

Currently, none of the known coronavirus protein receptors, including angiotensin-converting enzyme 2 (5, 6), aminopeptidase N (APN) (7, 8), dipeptidyl peptidase 4 (9) and carcinoembryonic antigen-related cell adhesion molecule 1 (10), are recognized as functional receptors for PEDV entry. Although earlier studies suggested that APN is a functional receptor for PEDV entry (3, 11–16), recent publications have raised doubts about this claim (7, 8, 17–23). For example, PEDV-permissive Vero cells lack APN protein expression (18). MDCK and HeLa cells that overexpress porcine aminopeptidase N (pAPN), as well as pAPN-positive porcine CPK cells, are more susceptible to transmissible gastroenteritis virus (TGEV) than to PEDV (7, 8). Moreover, pAPN-knockout (KO) pigs are resistant to TGEV but not to PEDV (21, 22). Recent studies have identified several host sugars or proteins that facilitate PEDV entry. Sialic acid and heparan sulfate serve as attachment factors to promote PEDV invasion during the initial stages of infection (3, 24–26). hDC-SIGN/L-SIGN or pDC-SIGN can mediate PEDV entry and propagation in PEDV-resistant cell lines (27). Occludin, a tight junction protein, is essential for PEDV infection at the postbinding stage (28). Moreover, integrins also play roles in PEDV entry. Specifically, integrin αvβ3 has been shown to increase PEDV replication (29). Additionally, the death receptor DR5, also known as tumor necrosis factor receptor superfamily member 10b, promotes PEDV invasion at an early stage by regulating caspase-8-dependent apoptosis (30). Furthermore, PEDV can activate the epidermal growth factor receptor, a transmembrane tyrosine receptor that augments PEDV infection and suppresses the host antiviral response via STAT3-mediated signaling (31). Moreover, the PEDV S1 protein interacts with the extracellular region of transferrin receptor 1 (TfR1) to facilitate PEDV entry by activating TfR1 tyrosine phosphorylation mediated by Src kinase (32). Although PEDV primarily infects villous enterocytes of the porcine small intestine, it also can infect cells from various species, including humans, monkeys, bats, and rats (3, 33). These findings suggest that PEDV exploits evolutionarily conserved cellular components as receptors, indicating a potential threat to other species. Understanding the molecules used for PEDV entry is crucial for gaining insights into its host range, cellular tropism, and evolutionary origins. This knowledge is not only fundamental to virology but also vital for evaluating the potential risk posed by PEDV to other species, particularly humans.

Genome-wide CRISPR/Cas9 screening is a powerful technique used to analyze the complex interactions between hosts and viruses. It has increasingly been applied to identify the host factors involved in the viral life cycle. Numerous host factors have been identified that either facilitate or inhibit the replication of various viruses, including severe acute respiratory syndrome coronavirus 2 (SARS-CoV-2), HIV-1, TGEV, Japanese encephalitis virus, PEDV, Ebola virus, chikungunya virus, enterovirus B, alphaviruses, and porcine deltacoronavirus (34–42). In this study, we employed a genome-wide CRISPR/Cas9 screen to identify host factors essential for PEDV entry. Our findings show that PEDV exploits interferon-inducible transmembrane (IFITM) proteins to facilitate its entry into host cells. Specifically, our results revealed that human IFITM3 enhances PEDV entry and replication in human cells, challenging the traditional view that IFITMs act solely as antiviral factors.

Furthermore, our results highlight the differential roles of porcine IFITMs. We observed that porcine IFITM1 significantly increased PEDV infection in both porcine cell lines and small intestinal organoids, whereas IFITM2 and IFITM3 did not have similar effects. These findings offer new insights into the molecular dynamics of PEDV entry, suggesting that IFITM proteins play diverse roles depending on the species and specific IFITM members. Overall, our study illuminates the role of human and porcine IFITMs in PEDV infection and highlights the crucial need to explore these interactions further. Understanding these dynamics is essential for assessing the risk of PEDV and similar viruses crossing species barriers, emphasizing the potential for these factors to influence broader host-virus interactions.

## RESULTS

### Genome-wide CRISPR/Cas9 screening identifies human IFITM3 as a critical host factor for PEDV infection

**Fig 1:**
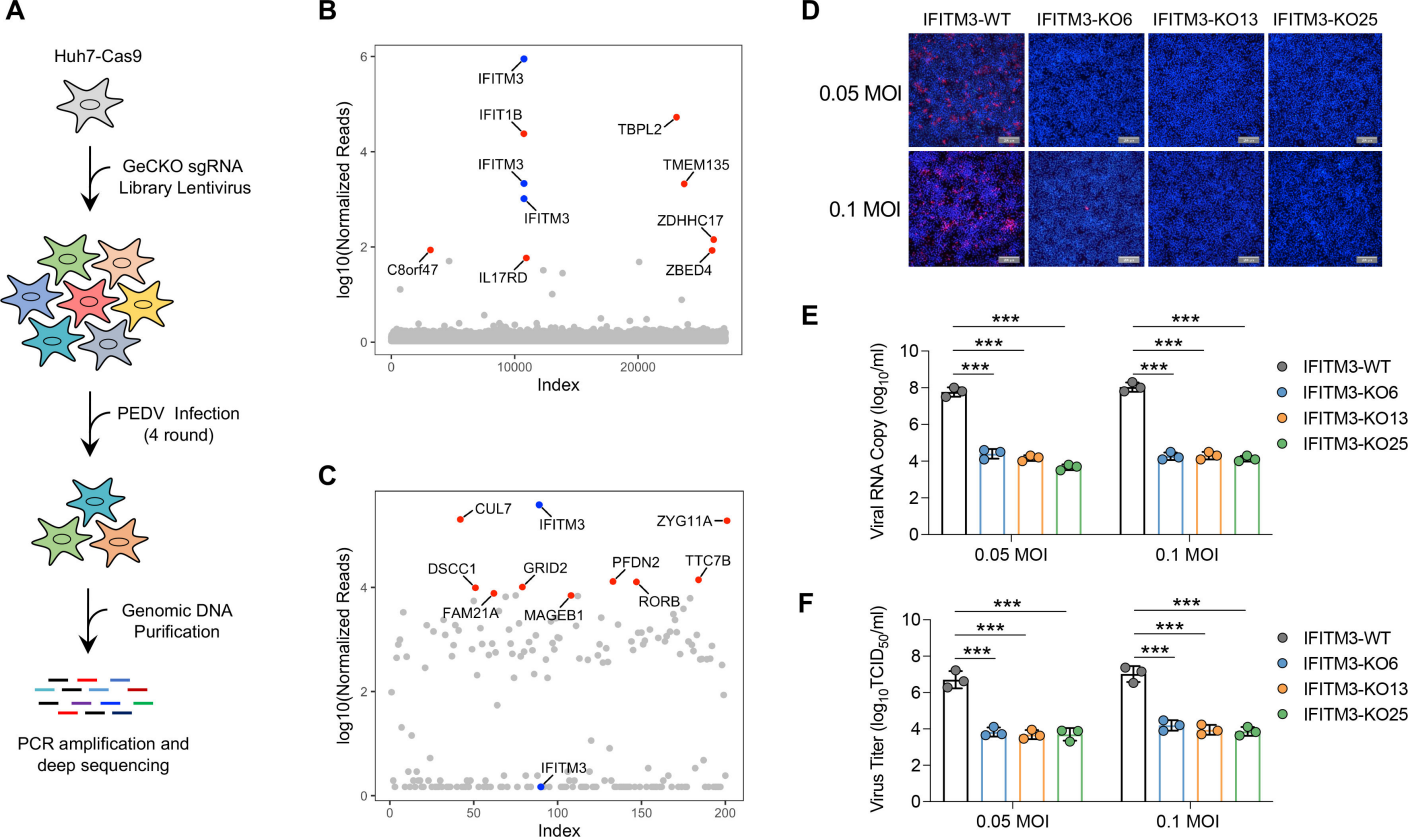
Identification of host factors essential for PEDV infection via genome-wide CRISPR/Cas9 screening. (**A**) Overview of the genome-wide CRISPR/Cas9 screening process conducted in Huh7 cells. Cas9-expressing Huh7 cells were transduced with a genome-wide sgRNA lentiviral library and then infected with PEDV at an MOI of 0.01 or 0.1. Surviving cells were harvested, and sgRNAs were amplified via PCR, followed by quantification of their abundance via next-generation sequencing. The sgRNA abundance from the genome-wide CRISPR/Cas9 screen is shown for infections with 0.01 MOI (**B**) or 0.1 MOI (**C**) of PEDV. The *x*-axis indicates the number of sgRNAs, whereas the *y*-axis represents the log_10_ value of the normalized sgRNA reads. The 10 most enriched sgRNAs are highlighted in blue and red for clarity. (**D**) IFITM3-wild-type (WT) and IFITM3-KO Huh7 cells were inoculated with PEDV SD at an MOI of 0.05 or 0.1. At 12 h post-infection, the cells were fixed and visualized via immunofluorescence staining with a mouse monoclonal antibody targeting the PEDV nucleocapsid (**N**) protein (red). The cell nuclei were stained with DAPI (blue). Scale bar: 200 µm. (**E**) The viral RNA in the supernatants was quantified by quantitative RT-PCR and is presented as the viral RNA copy number per milliliter. The error bars indicate the standard deviations (s.d.) of three biological replicates (*n* = 3). (**F**) Infectious PEDV particles in the supernatants of IFITM3-WT and -KO Huh7 cells were assessed via the TCID_50_ assay. The error bars indicate the s.d. from three independent experiments. ***, *P* < 0.001.

Understanding the role of cellular factors in PEDV infection is crucial for developing effective treatment strategies and vaccines against this devastating viral disease. To identify the host factors required for PEDV infection, we conducted a genome-wide CRISPR/Cas9 loss-of-function screen via a lentiviral small guide RNA (sgRNA) library. The screen was conducted in Huh7 cells (3, 33), which stably express the Cas9 protein (Fig. S1A). These cells were transduced with lentiviruses that express GeCKO v2 human lentiviral sgRNA library module B, which targets 19,050 protein-coding genes with three sgRNAs per gene. Lentiviral transduction was performed at a multiplicity of infection (MOI) of 0.3 to ensure that only one sgRNA virus infected each transduced cell. Sufficient cells were transduced to achieve approximately 1,000-fold coverage of each sgRNA in the library. Following transduction, the cells were selected for stable viral integration with puromycin. The cells subsequently underwent PEDV infection for four consecutive rounds to generate a pool of PEDV-resistant cells. Two independent pools were obtained via infection with the PEDV SD strain (43) at either 0.01 MOI or 0.1 MOI (Fig. S1B). Finally, genomic DNA was extracted from the surviving cells of both independent pools. The integrated sgRNA cassettes were amplified from the genomic DNA via PCR and subjected to next-generation sequencing (Fig. 1A).

The abundances of sgRNAs in the two independent screens were quantified via the logarithm (base 10) of the normalized counts for each sgRNA. The results revealed that the top 10 sgRNAs enriched in the first screen targeted IFITM3, TBPL2, ZDHHC17, CLCN3, IFIT1B, SETX, TMEM135, and MANSC1. Notably, the sgRNA targeting IFITM3 was the most enriched, with all three sgRNAs against IFITM3 enriched (Fig. 1B). Similarly, in the second screen, the top 10 enriched sgRNAs targeted IFITM3, CUL7, ZYG11A, TTC7B, PFDN2, GRID2, DSCC1, FAM21A, MAGEB1, and GNB4, with the sgRNA against IFITM3 again being the most enriched and two of the three sgRNAs targeting IFITM3 being enriched (Fig. 1C). We then transduced Huh7 cells with a lentiviral vector expressing the Cas9 protein and sgRNA targeting the IFITM3 gene and obtained three monoclonal IFITM3-knockout cell lines (IFITM3-KO6, IFITM3-KO13, and IFITM3-KO25) (Fig. S1C). The IFITM3-KO and IFITM3-wild-type (WT) Huh7 cell lines were inoculated with the PEDV SD strain. The expression of the PEDV N protein within infected cells was assessed via Western blot (Fig. S1D and E) and immunofluorescence (Fig. 1D; Fig. S1F) assays. Compared with those in IFITM3-WT cells, the intracellular levels of the viral N protein in IFITM3-KO cells were significantly lower. Viral RNA levels and virus titers in the supernatant were subsequently verified via RT-qPCR and 50% tissue culture infectious dose (TCID_50_) assays, respectively. Consistent with the reduction in viral N protein levels, both the viral RNA levels (Fig. 1E) and the production of progeny virions (Fig. 1F) were significantly decreased in IFITM3-KO cells. Collectively, these results indicate that IFITM3 plays a critical role in PEDV infection.

### Endogenous expression of the IFITM3 protein promotes PEDV infection

**Fig 2:**
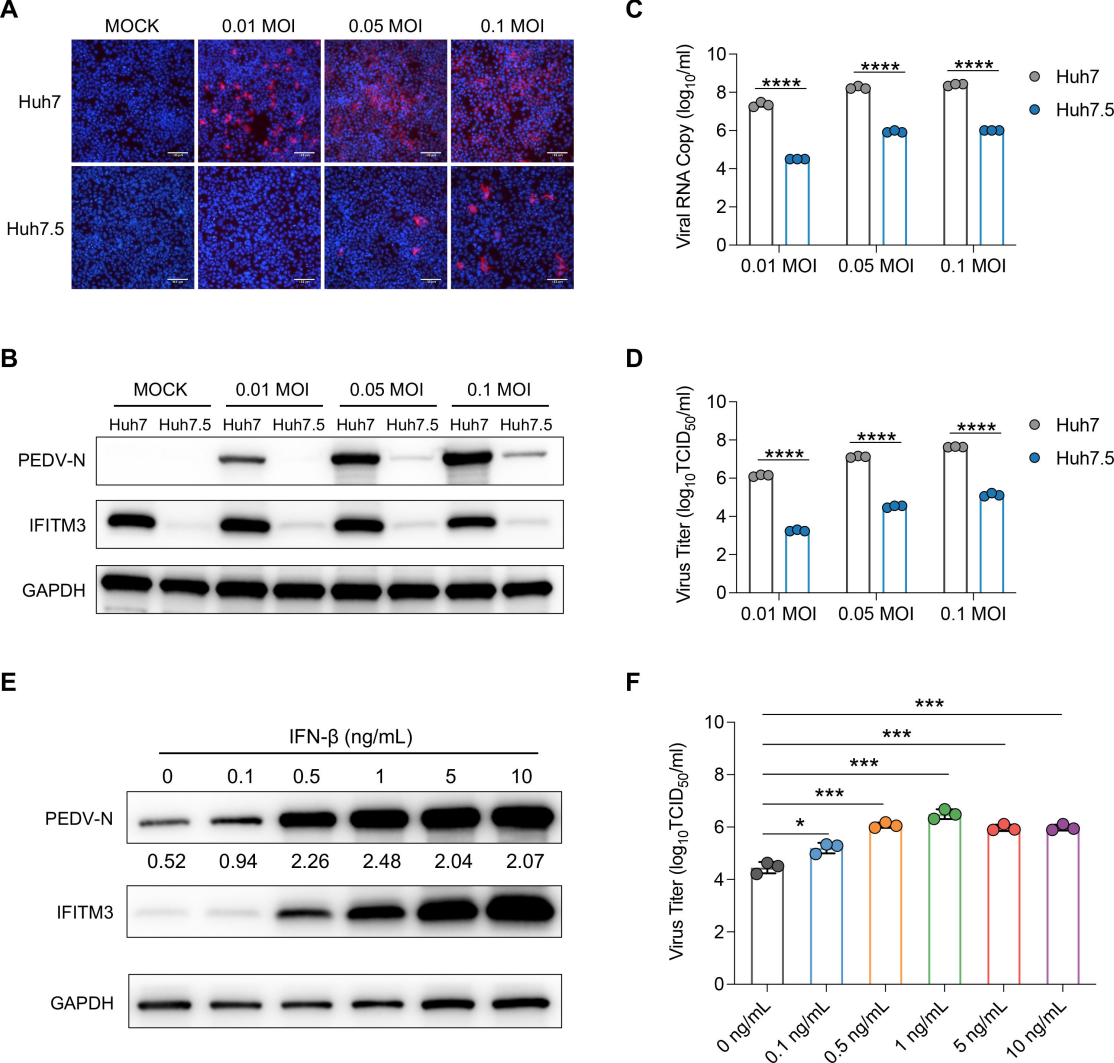
Endogenous IFITM3 expression enhances PEDV infection. (**A**) Huh7 and Huh7.5 cells were inoculated with the PEDV SD virus at MOIs of 0.01, 0.05, and 0.1. At 12 h post-infection, the cells were fixed and visualized via immunofluorescence staining. Representative fluorescence microscopy images of the intracellular PEDV N protein in Huh7 and Huh7.5 cells. Red indicates the viral N protein, and blue denotes the cell nuclei (DAPI). Scale bar: 200 µm. (**B**) Western blot analysis revealed the protein levels of PEDV N protein and IFITM3 in both the Huh7 and Huh7.5 cell lines after infection. GAPDH served as an internal control. (**C**) The viral genomic RNA in the supernatants was quantified via qRT-PCR and is presented as the viral RNA copy number per milliliter. The error bars indicate the s.d. from three technical replicates (*n* = 3). ****, *P* < 0.0001. (**D**) The number of infectious PEDV particles in the supernatants of Huh7 and Huh7.5 cells infected with different MOIs of PEDV SDs was assessed via the TCID_50_ assay. The error bars indicate the s.d. from three technical experiments. ****, *P* < 0.0001. (**E**) The protein expression of PEDV N protein and IFITM3 in Huh7.5 cells treated with different doses of IFN-β was analyzed by Western blotting. The relative expressions of the PEDV N protein were normalized against GAPDH, as indicated by the numerical values displayed below the blot. (**F**) Huh7.5 cells were treated with various doses of IFN-β for 12 h and then infected with 0.01 MOI of PEDV. Virus titers in the supernatant were analyzed via the TCID_50_ assay. *, *P* < 0.05; ***, *P* < 0.001.

Compared with Huh7 cells, the Huh7.5 cell line, a subclone of Huh7 cells, is characterized by lower expression levels of IFITM3 protein (44). We first confirmed this difference through Western blot analysis with an IFITM3 monoclonal antibody (Fig. S2A). To further investigate the impact of variations in IFITM3 expression on PEDV infection efficiency, we infected both Huh7 and Huh7.5 cells with different MOIs (0.01, 0.05, and 0.1) of the PEDV SD strain. We then assessed the level of the intracellular PEDV N protein in the infected cells via both an immunofluorescence assay (Fig. 2A; Fig. S2B) and a Western blot (Fig. 2B; Fig. S2C). The results indicated that the intracellular levels of the viral N protein in Huh7.5 cells were significantly lower than those in Huh7 cells. Additionally, we collected the supernatant from the infected cells to quantify viral RNA copy numbers (Fig. 2C) and to assess the production of progeny virions via the TCID_50_ assay (Fig. 2D). Consistent with the viral N protein levels, the viral RNA copy number and viral titer in Huh7.5 cells were significantly lower than those in Huh7 cells.

IFITM3 is a transmembrane protein induced by IFNs. To further investigate whether the endogenous IFITM3 induced by IFNs facilitates PEDV infection, we assessed the impact of IFN-β on viral infection in Huh7.5 cells. Our analysis revealed that IFN-β treatment enhanced PEDV infection in Huh7.5 cells in a dose-dependent manner, as evidenced by a significant increase in the intracellular N protein level (Fig. 2E) and progeny virion yield (Fig. 2F). However, IFN-β only enhanced PEDV replication at a low dose and peaked at a concentration of 1 ng/mL in Huh7.5 cells (Fig. S2C). As the concentration of IFN-β increased, its ability to promote PEDV replication decreased despite a continuous increase in IFITM3 expression (Fig. 2E). These findings suggest that the endogenous expression of the IFITM3 protein facilitates PEDV infection.

### Forced expression of the human IFITM3 or IFITM2 protein promotes PEDV infection

**Fig 3:**
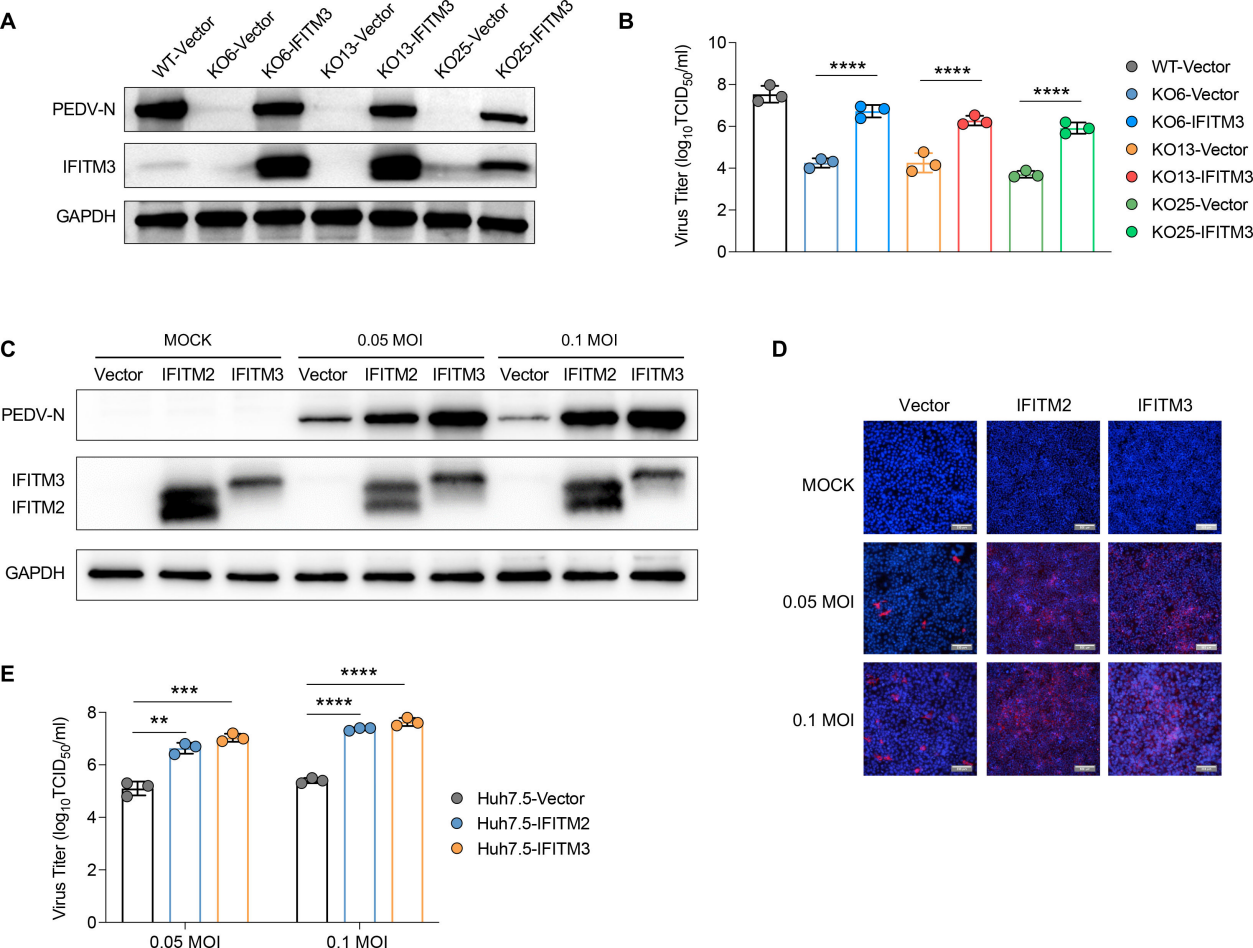
Both IFITM2 and IFITM3 enhance PEDV infection. (**A**) IFITM3 was reintroduced into IFITM3-KO Huh7 cells via transduction with a lentivirus expressing IFITM3 or a control empty vector, after which the cells were infected with the PEDV SD strain. At 24 h post-infection, the levels of the IFITM3 protein and PEDV N protein were analyzed via Western blotting. (**B**) The viral titers in the supernatants of the various cells in (**A**) were assessed via the TCID_50_ assay. ****, *P* < 0.0001. (**C**) Huh7.5 cells were transduced with lentiviral vectors expressing either IFITM2 or IFITM3 or a control empty vector, followed by PEDV infection. The expression of the PEDV N protein and IFITM protein was assessed by Western blot analysis. (**D**) The expression of the PEDV N protein from (**C**) was also visualized via immunofluorescence staining. Representative images of the intracellular PEDV N protein. Red indicates the viral N protein, and blue denotes the cell nuclei (DAPI). Scale bar: 200 µm. (**E**) Huh7.5 cells were transduced with lentiviral vectors expressing either IFITM2 or IFITM3 or a control empty vector, followed by PEDV infection. The viral titers in the supernatant were measured via the TCID_50_ assay. **, *P* < 0.01; ***, *P* < 0.001; ****, *P* < 0.0001.

We demonstrated that the knockout of IFITM3 inhibits PEDV infection. To exclude the possibility of off-target effects, we reintroduced the IFITM3 gene into IFITM3-KO Huh7 cells to investigate whether the re-expression of IFITM3 restored the susceptibility of these cells to PEDV infection. IFITM3 re-expression was achieved by transducing IFITM3-KO Huh7 cells with a lentivirus expressing the IFITM3 protein (Fig. 3A). Our results revealed partial restoration of PEDV infectivity in IFITM3-KO Huh7 cells upon IFITM3 re-expression, as assessed by measuring the intracellular levels of the viral N protein (Fig. 3A) and evaluating the yields of progeny virions (Fig. 3B). Specifically, we observed that compared with control cells, IFITM-KO cells re-expressing IFITM3 exhibited a significant increase in PEDV infection. However, these cells presented lower viral titers than IFITM-WT Huh7 cells did. These results indicate that IFITM3 overexpression partially restores PEDV infection in IFITM3-KO cells, suggesting that IFITM3 plays a critical role in the PEDV infection process.

To further investigate the role of IFITM proteins in PEDV infection, we overexpressed IFITM3 and two other IFITM family members, IFITM1 and IFITM2, in Huh7.5 cells by transducing them with a lentiviral vector expressing IFITM proteins (Fig. 3C; Fig. S3A). These cells were subsequently infected with the PEDV SD strain to assess the impact of the IFITM proteins on PEDV infection. The results demonstrated that the expression of either the IFITM2 or the IFITM3 protein significantly enhanced PEDV infection, as evidenced by substantial increases in the intracellular levels of the viral N protein (Fig. 3C and D) and yields of progeny virions (Fig. 3E). In contrast, although IFITM1 slightly increased during PEDV infection (Fig. S3B through D), this effect was not statistically significant. These findings indicate that IFITM3 and IFITM2 facilitate PEDV infection in Huh7.5 cells.

### IFITM3 protein facilitates PEDV entry into host cells

**Fig 4:**
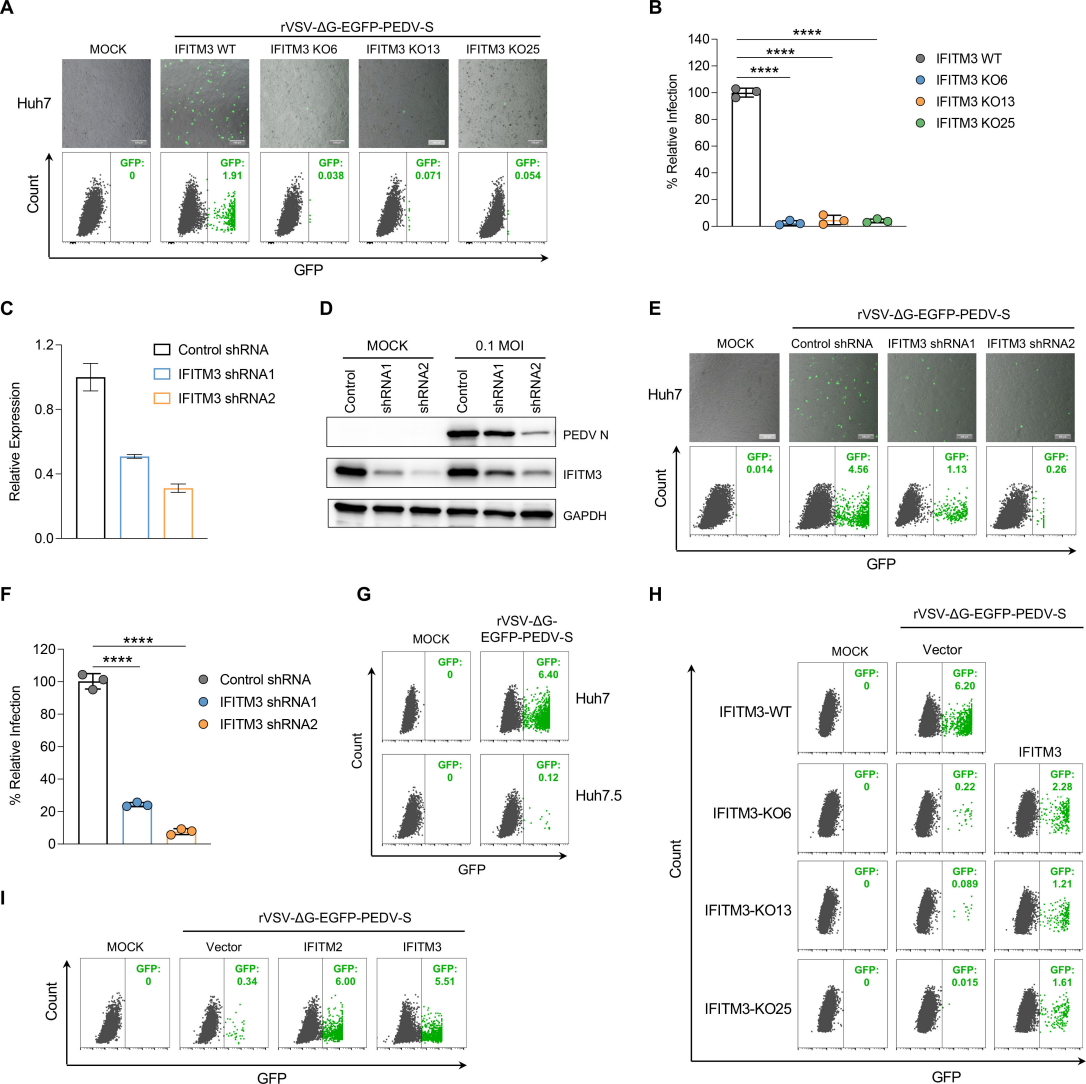
IFITM3 enhances PEDV entry into host cells. (**A**) IFITM3-WT and IFITM3-KO Huh7 cells were infected with equal amounts of the PEDV spike pseudotyped virus rVSV-ΔG-EGFP-PEDV-S. Samples were collected at 12 h post-infection (hpi) and analyzed via fluorescence microscopy and flow cytometry. Scale bars: 200 µm. (**B**) The relative transduction rates obtained from the flow cytometry analysis in (**A**) were quantified. The data are expressed as the means ± s.d. of three independent experiments. ****, *P* < 0.0001. (**C**) RT-qPCR data showing the relative expression of IFITM3 mRNA in Huh7 cells transduced with lentiviral vectors carrying either IFITM3 shRNAs or scramble shRNAs (control). IFITM3 expression in control cells was normalized to 1. The values are presented as the means ± s.d., *n* = 3. (**D**). Representative Western blots showing the levels of the PEDV N protein (top), IFITM3 protein (middle), and GAPDH (bottom; loading control) in Huh7 cells transduced with lentiviral vectors containing IFITM3 shRNAs or scramble shRNA. (**E**) IFITM3-knockdown cells were infected with the same amount of rVSV-ΔG-EGFP-PEDV-S. At 12 hpi, the efficiency of virus transduction was assessed via fluorescence microscopy and flow cytometry. (**F**) The relative transduction rates of rVSV-ΔG-EGFP-PEDV-S in IFITM3-knockdown Huh7 cells were analyzed via flow cytometry. The values are presented as the means ± s.d. (*n* = 3). ****, *P* < 0.0001. (**G**) A comparison of the transduction rates between Huh7 and Huh7.5 cells infected with equal amounts of rVSV-ΔG-EGFP-PEDV-S is shown. (**H**) Flow cytometry analysis of IFITM3-KO Huh7 cells re-expressing IFITM3 and infected with the same amount of rVSV-ΔG-EGFP-PEDV-S. (**I**) Flow cytometry analysis of Huh7.5 cells overexpressing either IFITM3 or IFITM2 and infected with equal amounts of rVSV-ΔG-EGFP-PEDV-S. All data are presented as the means ± s.d. from three independent experiments.

Considering the predominant presence of the IFITM3 protein in endosome and lysosome membranes, we hypothesized that it acts as a host factor facilitating the entry of PEDV rather than merely serving as a restriction factor for other viruses. To test this hypothesis, we used a replication-deficient vesicular stomatitis virus (VSV) named rVSV-ΔG-EGFP-PEDV-S, in which the glycoprotein gene was replaced with the EGFP gene and the VSV glycoprotein was substituted with the PEDV spike protein (45). Both IFITM3-WT and IFITM3-KO cells were incubated with either rVSV-ΔG-EGFP-PEDV-S or rVSV-ΔG-EGFP-G as a control, and virion entry into the cells was assessed via fluorescence microscopy and flow cytometry. The results revealed a significant decrease in rVSV-ΔG-EGFP-PEDV-S entry into IFITM3-KO cells compared with IFITM3-WT cells (Fig. 4A and B). In contrast, no significant difference was observed between IFITM3-KO cells and IFITM3-WT cells infected with the rVSV-ΔG-EGFP-G virus (Fig. S4A). We also evaluated the entry efficiency of VSV pseudotyped with Middle East respiratory syndrome coronavirus (MERS-CoV) and SARS-CoV-2 spike proteins in IFITM3-KO cells. As expected, the absence of IFITM3 led to an increase in the entry efficiency of both MERS-CoV and SARS-CoV-2 pseudoviruses compared with that of IFITM3-WT cells (Fig. S4B).

To further confirm the role of IFITM3 in PEDV entry, we knocked down IFITM3 in Huh7 cells via lentiviral vectors expressing short hairpin RNAs (shRNAs) targeting IFITM3 (Fig. 4C and D). We examined PEDV entry in IFITM3-knockdown Huh7 cells by infecting them with rVSV-ΔG-EGFP-PEDV-S. The results revealed a significant reduction in the percentage of green fluorescent protein (GFP)-positive cells in the IFITM3-knockdown groups compared with the control shRNA group (Fig. 4E and F). Additionally, we evaluated viral replication by measuring the intracellular levels of the viral N protein and progeny virion yields. Consistent with the reduction in PEDV entry, both the intracellular levels of the viral N protein and the virus titers were significantly lower in IFITM3-knockdown Huh7 cells than in control shRNA-treated Huh7 cells (Fig. 4D; Fig. S4C). In contrast, no significant difference was observed between IFITM3-knockdown cells and IFITM3-WT cells infected with the rVSV-ΔG-EGFP-G virus (Fig. S4E).

We also compared the impact of IFITM3 on PEDV entry in Huh7 and Huh7.5 cells by infecting both cell lines with rVSV-ΔG-EGFP-PEDV-S. Notably, Huh7 cells presented a significantly greater number of GFP-positive cells than Huh7.5 cells did, indicating that IFITM3 enhances PEDV entry (Fig. 4G). Moreover, re-expression of IFITM3 in IFITM3-KO Huh7 cells partially restored PEDV entry into Huh7 cells (Fig. 4H), whereas overexpression of IFITM3 or IFITM2 in Huh7.5 cells increased PEDV entry (Fig. 4I). We assessed the infectivity of VSV pseudotyped with MERS-CoV or SARS-CoV-2 spike proteins in Huh7.5 cells overexpressing IFITM3 or IFITM2. The results demonstrated that overexpression of IFITM3 or IFITM2 in Huh7.5 cells effectively inhibited the entry of both MERS-CoV and SARS-CoV-2 (Fig. S4F). Finally, we treated Huh7.5 cells with various concentrations of IFN-β for 12 h to assess its effect on PEDV entry. The results revealed an increase in the percentage of GFP-positive cells at lower concentrations of IFN-β, with slight inhibition observed at higher concentrations (Fig. S4G). Collectively, these findings strongly support the role of IFITM3 in facilitating the entry of PEDV.

### Human IFITM3 facilitates the entry of PEDV after internalization

**Fig 5:**
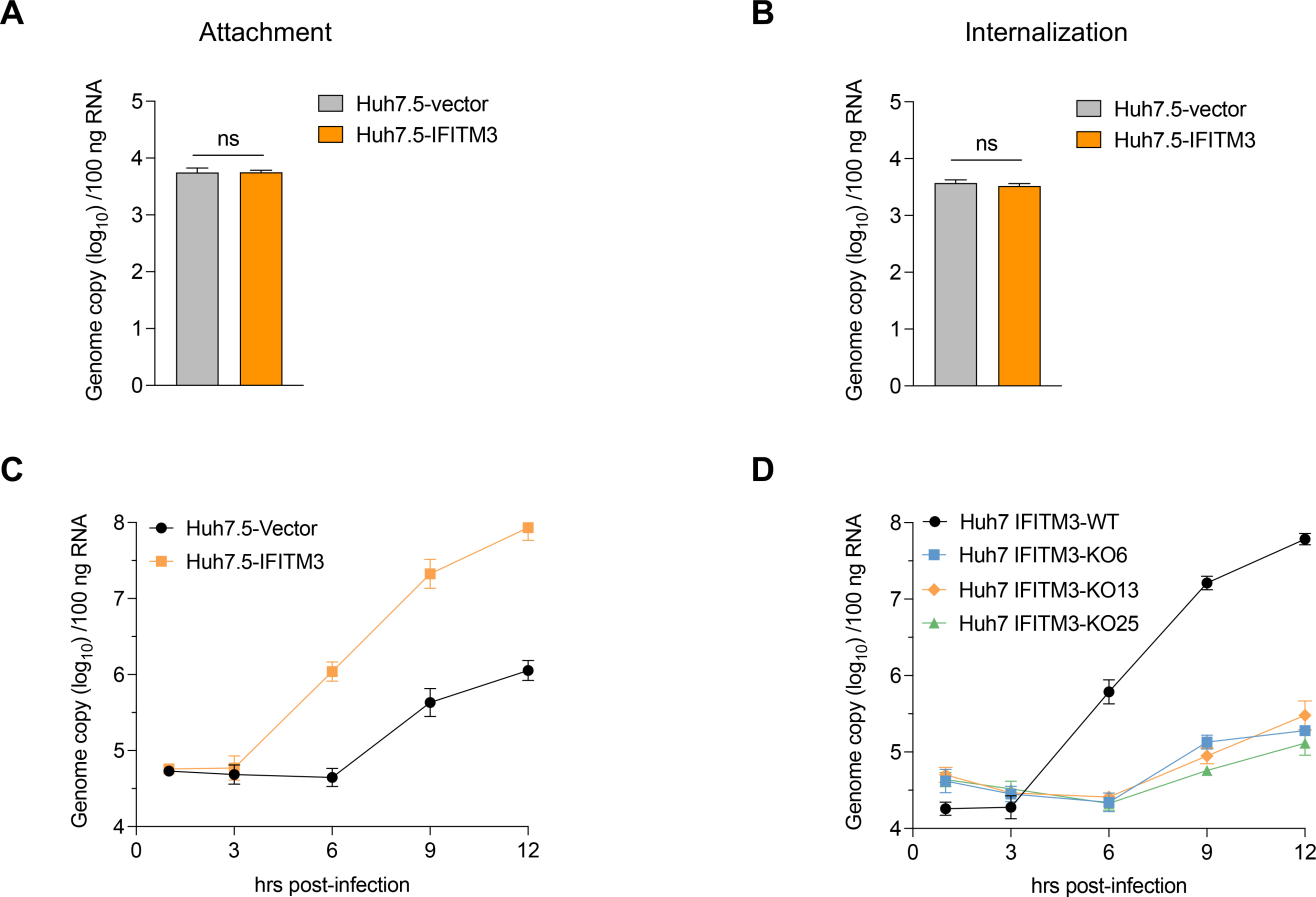
Impact of IFITM3 on PEDV attachment, internalization, and viral RNA replication. (**A**) Huh7.5-IFITM3 and Huh7.5-vector cells were infected with PEDV at an MOI of 10 for 1 h on ice. The cells were subsequently washed three times with cold DPBS, and viral attachment to the cell surface was quantified via RT-qPCR. (**B**) Following viral attachment for 1 h on ice, the cells were incubated at 37°C for an additional 1 h and viral internalization was assessed by RT-qPCR. (**C**) Huh7.5-IFITM3 and Huh7.5-vector cells, as well as (**D**) IFITM3-WT and IFITM3-KO cells, were infected with PEDV at an MOI of 10 at 37°C for 1 h. After the inoculum was removed, the cells were either immediately harvested or cultured at 37°C for the specified time points. The amounts of cell-associated viral RNA were quantified via a qRT-PCR assay and are presented as the copy number per 100 ng of total RNA. The data are presented as the means ± s.d. from three independent experiments.

To further investigate the entry mechanisms facilitated by IFITM proteins, we first assessed the effects of IFITM3 expression on the attachment and internalization of PEDV. The results indicated that the expression of human IFITM3 in Huh7.5 cells did not enhance either the attachment or internalization of PEDV (Fig. 5A and B). We subsequently evaluated the effect of IFITM3 overexpression on PEDV entry by infecting both Huh7.5 cells overexpressing IFITM3 and those transduced with an empty vector as a control. The impact of IFITM3 on PEDV entry was assessed by measuring the intracellular levels of the viral genome at various time points post-infection. Interestingly, viral RNA amplification was undetectable at 6 h post-infection (hpi) in control Huh7.5 cells. However, with IFITM3 expression, there was a noticeable reduction in the time required for viral RNA amplification by at least 3 h (Fig. 5C). Consistent with these findings, we further demonstrated that, compared with IFITM3-WT Huh7 cells, IFITM3-KO Huh7 cells presented a delay in viral RNA amplification, again by at least 3 h (Fig. 5D). On the basis of these experimental results, our findings suggest that IFITM3 plays a crucial role in facilitating PEDV entry during the later stages after internalization.

### Porcine IFITM1 protein enhances PEDV infection in LLC-PK1 cells

**Fig 6:**
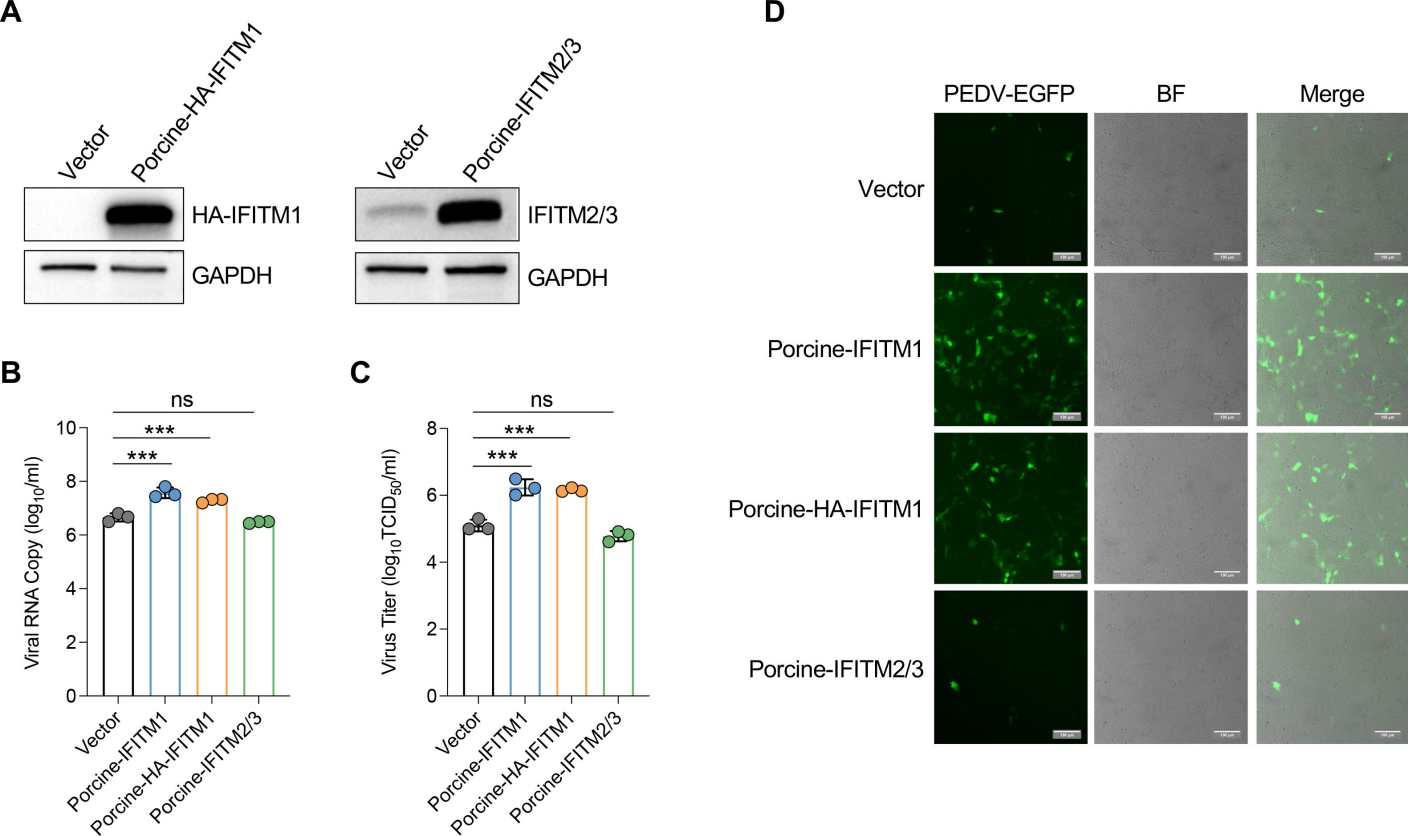
Porcine IFITM1 enhances PEDV infection in LLC-PK1 cells. (**A**) Western blot analysis of porcine IFITM1 and IFITM2/3 overexpression in LLC-PK1 cells transduced with lentiviral vectors expressing porcine HA-tagged porcine IFITM1, porcine IFITM2/3, or an empty vector as a negative control. Porcine IFITM1- or IFITM2/3-overexpressing LLC-PK1 cells were infected with the PEDV-HM strain. (**B**) Viral RNA copies in the supernatant were quantified by RT-qPCR and are presented as copy numbers. The data are presented as the means ± s.d. from three technical repeats. (**C**) Viral titers in the supernatant were measured via the TCID_50_ assay. The data are presented as the means ± s.d. from three independent experiments. (**D**) LLC-PK1 cells overexpressing porcine IFITM1 or IFITM2/3 were infected with rPEDV-HM-EGFP (0.1 MOI), and the EGFP-fluorescent cells were imaged via fluorescence microscopy at 24 hpi. The green cells represent PEDV-infected cells, while background cells were visualized via white light. Scale bar: 200 µm.

Compared with the human IFITM1/2/3 genes, the porcine genome contains two IFITM genes, as indicated by the *Sus scrofa* genome annotation from GenBank, designated IFITM1 and IFITM2/3 (Fig. S5). To evaluate the effects of porcine IFITMs on PEDV infection, we transduced LLC-PK1 cells with lentiviral vectors that expressed either porcine IFITM1 or IFITM2/3 proteins or an empty vector as a control. Additionally, we expressed hemagglutinin (HA)-tagged IFITM1 (IFITM1-HA) in LLC-PK1 cells to address the absence of a specific antibody for porcine IFITM1 (Fig. 6A). These cells were then inoculated with the PEDV-HM strain to observe the effects of IFITM expression on viral infection. Viral RNA levels (Fig. 6B) and progeny virion production (Fig. 6C) in the supernatant were analyzed by RT-qPCR and TCID_50_. The results showed that the expression of porcine IFITM1 or IFITM1-HA protein, but not IFITM2/3, significantly increased the infection of cells by PEDV-HM. To further validate these findings, we infected LLC-PK1 cells overexpressing IFITMs with a recombinant PEDV, designated rPEDV-HM-EGFP, which expresses the EGFP marker (33, 46). Consistent with the results obtained via PEDV-HM, we observed a significant increase in the GFP signal in LLC-PK1 cells overexpressing IFITM1 compared with that in control cells. In contrast, we observed a slight decrease in the GFP signal in cells overexpressing IFITM2/3 (Fig. 6D). Overall, these findings suggest that IFITM1, but not IFITM2/3, promotes PEDV infection in LLC-PK1 cells, indicating distinct roles for these proteins in the context of viral infectivity.

### The C-terminal residues are critical for enhancing PEDV entry in porcine IFITM2/3

**Fig 7:**
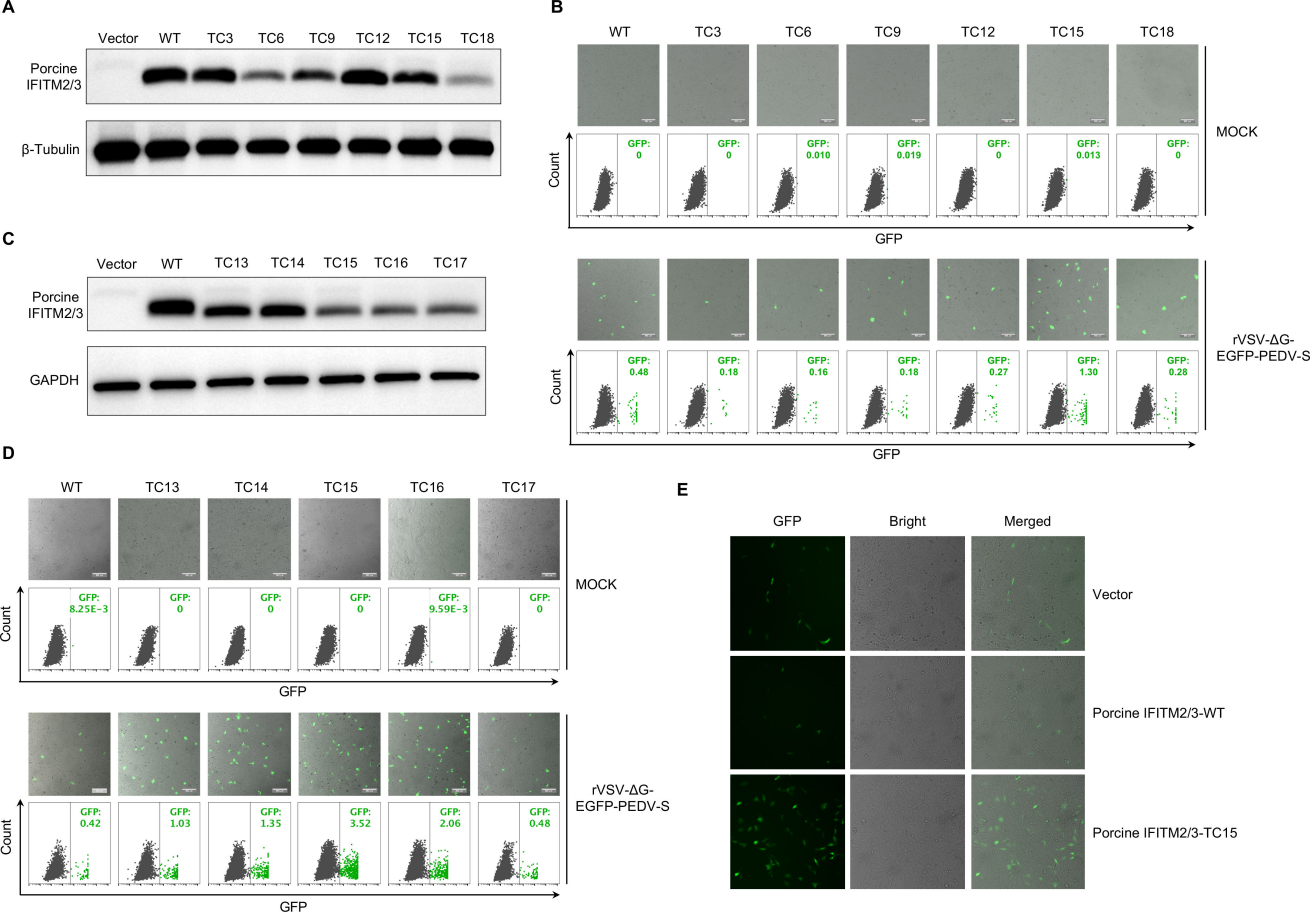
Effects of the C-terminus of IFITM2/3 on PEDV entry. (**A**) Huh7.5 cells were transduced with lentiviral vectors expressing various HA-tagged IFITM2/3 C-terminal truncations (WT, TC3, TC6, TC9, TC12, TC15, or TC18) or an empty vector as a control. The expression of HA-tagged IFITMs was detected via a Western blot analysis using an anti-HA monoclonal antibody. (**B**) Fluorescence microscopy images and corresponding flow cytometry analyses of Huh7.5 cells expressing IFITM2/3 C-terminal truncation variants and infected with rVSV-ΔG-EGFP-PEDV-S are shown. The upper panel shows mock-infected cells. The lower panel shows cells infected with the recombinant virus. GFP expression levels are indicated for each variant, with values reflecting the percentage of GFP-positive cells. (**C**) Western blot analysis showing the expression of porcine IFITM2/3 C-terminal truncation variants (WT, TC13, TC14, TC15, TC16, and TC17) in Huh7.5 cells. (**D**) Fluorescence microscopy images and flow cytometry analyses of additional IFITM2/3 C-terminal truncated variants following infection with rVSV-ΔG-EGFP-PEDV-S are shown. The upper panel shows mock-infected cells, whereas the lower panel presents virus-infected cells with the indicated GFP expression levels. (**E**) Porcine IFITM2/3-WT- or porcine IFITM2/3-TC15-overexpressing LLC-PK1 cells were infected with rPEDV-HM-EGFP (0.1 MOI), and the EGFP-fluorescent cells were imaged via fluorescence microscopy at 24 hpi. The green cells represent PEDV-infected cells, and the background cells were visualized under white light.

The C-terminus of porcine IFITM2/3 is similar to that of human IFITM1 (Fig. S5). Previous studies have shown that truncation of the C-terminus of human IFITM1 effectively promotes the entry of human coronavirus OC43 (44). On the basis of these findings, we hypothesized that removing amino acids (aa) from the C-terminus of porcine IFITM2/3 could facilitate PEDV entry. To test this hypothesis, we initially truncated 3, 6, 9, 12, 15, and 18 aa from the C-terminus of porcine IFITM2/3 and overexpressed these IFITM2/3 variants in Huh7.5 cells (Fig. 7A). We then infected these cells with the PEDV pseudovirus rVSV-ΔG-EGFP-PEDV-S. The entry of the pseudovirus into the cells was assessed via fluorescence microscopy and flow cytometry (Fig. 7B). The results indicated that the deletion of the C-terminal 15 aa residue of porcine IFITM2/3 increased PEDV entry into Huh7.5 cells. We subsequently truncated 13, 14, 16, and 17 aa from the C-terminus of porcine IFITM2/3 (Fig. 7C). The results indicated that truncating by 13, 14, and 16 amino acids resulted in increased entry of PEDV into Huh7.5 cells (Fig. 7D). Furthermore, we overexpressed IFITM2/3 with a 15 aa truncation in LLC-PK1 cells. Consistent with the results obtained from experiments conducted in Huh7.5 cells, the deletion of the 15 C-terminal aa residues significantly enhanced PEDV infection in LLC-PK1 cells (Fig. 7E). These findings suggest that the C-terminal regions of IFITM2/3 are critical for modulating the entry of PEDV into host cells.

### Interaction and colocalization of the PEDV S protein with IFITM proteins

**Fig 8:**
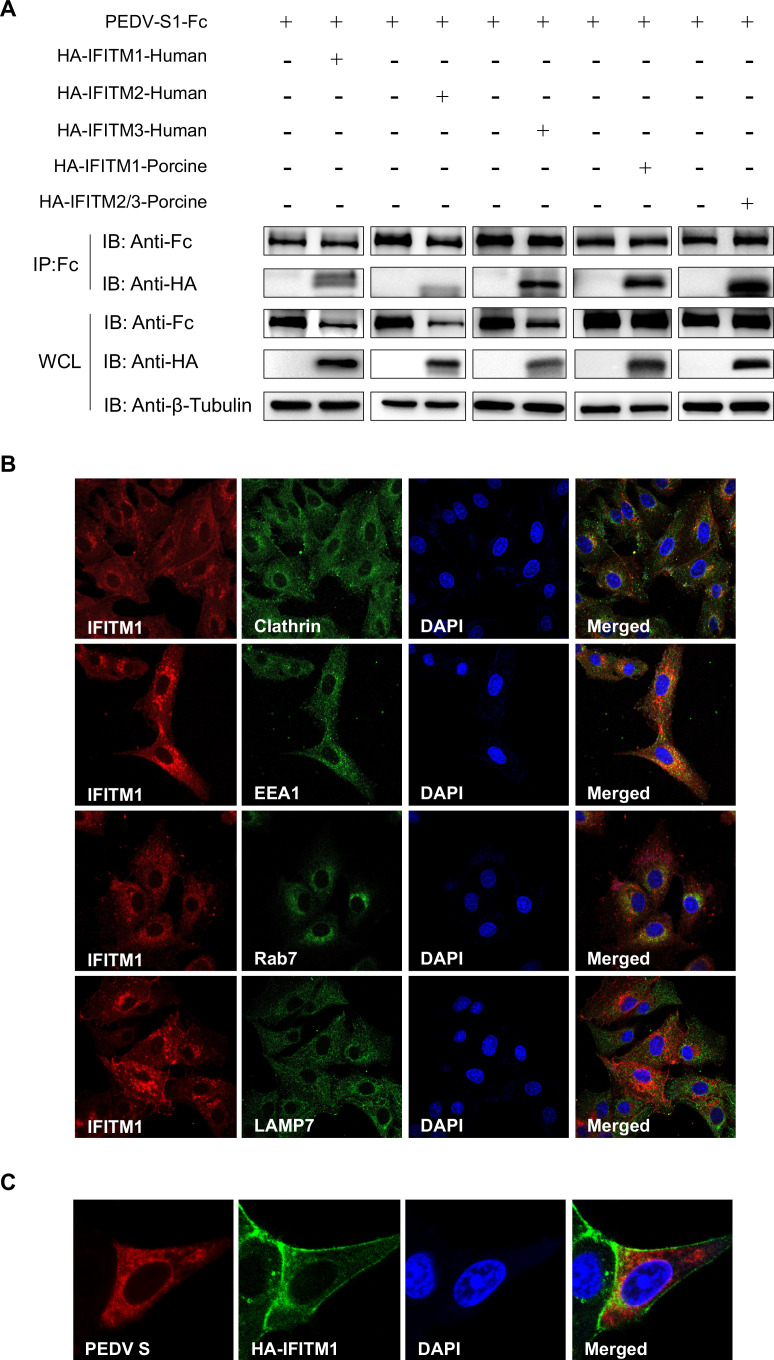
Interaction and colocalization of the PEDV S protein with IFITM proteins. (**A**) Co-IP assays were performed to assess the interaction between the PEDV S1 protein and human or porcine IFITM proteins. HEK293T cells were cotransfected with plasmids encoding PEDV S1-Fc and various HA-tagged IFITM proteins. At 24 h post-transfection, the cells were harvested. Immunoprecipitation was conducted using an Fc tag antibody, and the presence of IFITM proteins was detected by Western blotting (IB: Anti-HA). Whole-cell lysates (WCLs) were analyzed to confirm expression levels (IB: Anti-Fc, Anti-HA, Anti-β-Tubulin). The Co-IP was repeated three times, yielding similar results. (**B**) Confocal microscopy images showing the intracellular localization of porcine IFITM1 in LLC-PK1 cells are presented. IFITM1 (red) colocalizes with clathrin (green), EEA1 (green), Rab7 (green), and LAMP7 (green), as indicated in the merged images. DAPI (blue) was used to stain the nuclei. (**C**) Confocal microscopy images demonstrating the colocalization of the PEDV S protein (red) with HA-IFITM1 (green) in LLC-PK1 cells are shown. The merged image shows the nuclei stained with DAPI (blue).

The S protein of PEDV is a type I transmembrane glycoprotein. It consists of two distinct domains, namely S1 and S2, which play key roles in binding and fusion processes, respectively. To investigate the potential interaction between the PEDV S1 protein and IFITMs, coimmunoprecipitation (Co-IP) assays were performed. HEK293T cells were cotransfected with plasmids encoding the PEDV S1 protein along with either human or porcine IFITM1, IFITM2, or IFITM3. Following transfection, the cell lysates were subjected to immunoprecipitation with different tag mono-antibodies, and the presence of IFITM proteins in the immunoprecipitates was detected via Western blotting. Our results demonstrated that the PEDV S1 protein interacts with all IFITM proteins from both human and porcine origins (Fig. 8A; Fig. S6). The interaction was specific, as no IFITM proteins were detected in the immunoprecipitates from cells transfected with the control plasmid.

Porcine IFITM1 is a membrane protein consisting of 124 amino acids. Despite its potential significance in various biological processes, this protein has yet to be well-studied. We first investigated the intracellular localization of porcine IFITM1 in LLC-PK1 cells. Using confocal microscopy, we demonstrated that porcine IFITM1 colocalizes with clathrin, indicating its involvement in clathrin-mediated endocytosis. Furthermore, we observed that porcine IFITM1 is expressed in early endosomes, late endosomes, and/or lysosomes, suggesting its significant role in the endosomal pathway (Fig. 8B). To further explore the interaction between the PEDV S protein and porcine HA-IFITM1, we performed immunofluorescence assays in LLC-PK1 cells. The cells were cotransfected with plasmids expressing the PEDV S protein and porcine IFITM1. At 24 hpi, the cells were fixed, permeabilized, and stained with antibodies specific to the S protein and HA-IFITM1. Confocal microscopy revealed colocalization of the S protein with IFITM1 in the perinuclear region of the cells (Fig. 8C). These results indicate a direct interaction between the PEDV S1 protein and IFITM proteins in HEK293T cells, as well as significant colocalization of the PEDV S protein with porcine IFITM1 in LLC-PK1 cells. The interaction and colocalization findings suggest that IFITM proteins play a role in the life cycle of PEDV, potentially influencing viral entry.

### Porcine IFITM1 enhances PEDV infection in porcine small intestinal organoids (PSIOs)

**Fig 9:**
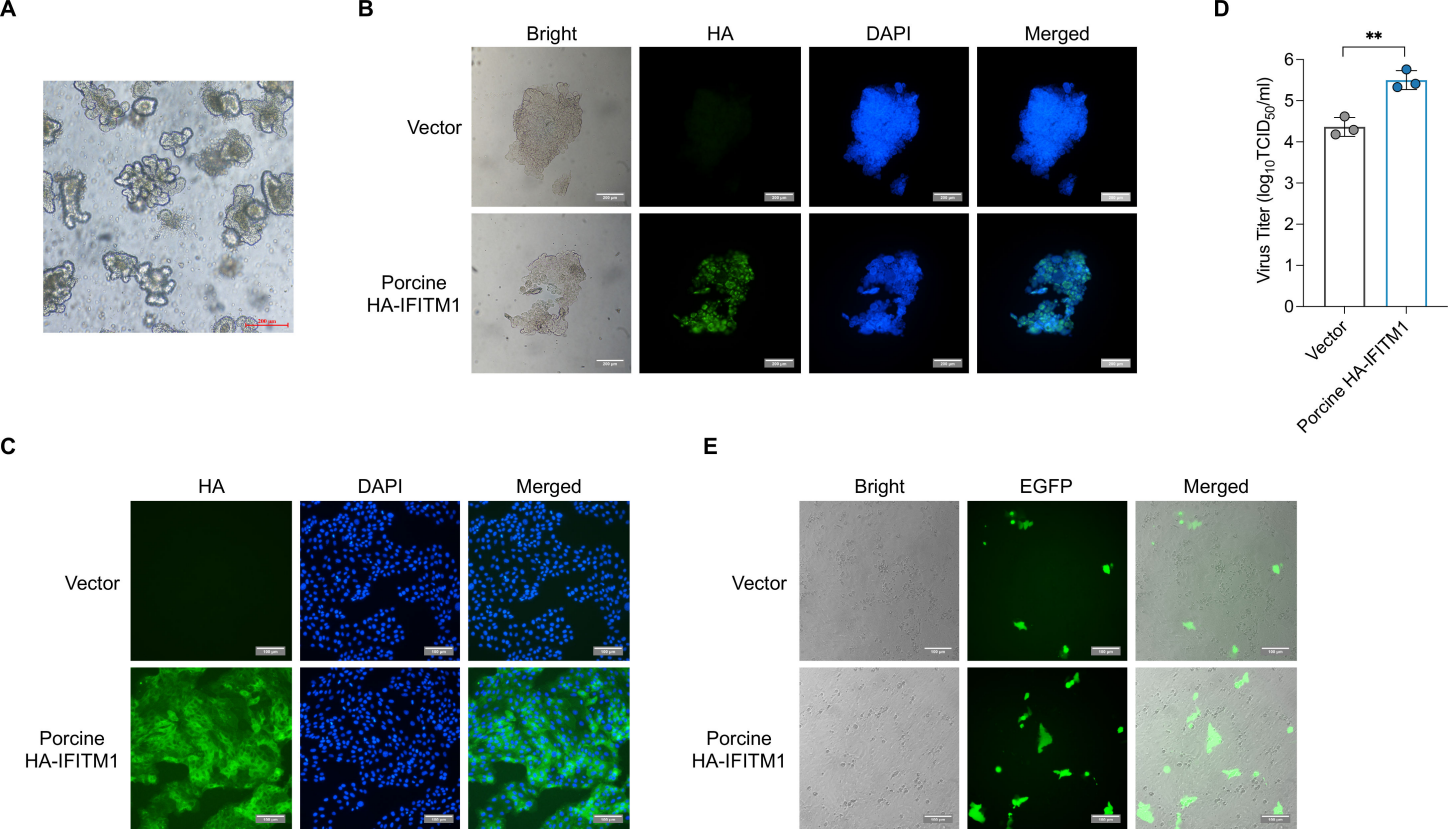
Porcine IFITM1 enhanced PEDV infection in porcine small intestinal organoids. (**A**) Porcine small intestinal crypts were isolated from neonatal piglets and cultured *in vitro*. A representative image depicting the morphology of porcine small intestinal organoids is shown. Scale bar: 200 µm. (**B**) Immunofluorescence confirmation of HA-tagged porcine IFITM1 overexpression in PSIOs. Representative images displaying IFITM1 (HA, green) and nuclei (DAPI, blue) are presented. (**C**) The expression of IFITM1 was verified via immunofluorescence in a 2D organoid culture system. Representative images showing HA (green) and nuclei (DAPI, blue) staining are presented. (**D**) Analysis of viral infection in IFITM1-overexpressing and control 2D organoids following PEDV infection is shown. IFITM1-overexpressing or control 2D organoids were infected with PEDV-HM (0.1 MOI). At 24 hpi, the virus titer in the supernatant was quantified according to the TCID_50_. The data are presented as the means ± s.d. from three independent experiments. **, *P* < 0.01. (**E**) IFITM1-overexpressing or control 2D organoids were infected with rPEDV-HM-EGFP (0.1 MOI), and the EGFP-fluorescent cells were imaged via fluorescence microscopy at 24 hpi. The green cells represent PEDV-infected cells, and the background cells were visualized under white light. Scale bar: 200 µm.

To further investigate the role of IFITM1 in PEDV infection, we overexpressed IFITM1 in PSIOs derived from neonatal piglets. The PSIOs were isolated and cultured as previously described (43). Initially, the PSIOs were cultured (Fig. 9A) and then transduced with a lentiviral vector expressing HA-IFITM1 or an empty vector control. The successful overexpression of IFITM1 in PSIOs was confirmed via immunofluorescence staining (Fig. 9B). A two-dimensional (2D) organoid culture system was subsequently established for both control and IFITM1-overexpressing organoids. The expression of IFITM1 in these 2D organoids was also verified via immunofluorescence staining (Fig. 9C) prior to infection with PEDV-HM.

Consequently, viral replication was assessed by the TCID_50_. The results revealed a significant increase in viral titers in IFITM1-overexpressing organoids compared with control organoids at 24 h hpi (Fig. 9D). The viral titers in IFITM1-overexpressing organoids were approximately 10-fold greater than those in control organoids. Moreover, we infected both control and IFITM1-overexpressing 2D organoids with rPEDV-HM-EGFP, a recombinant PEDV expressing EGFP derived from the PEDV-HM strain (46). Consistent with the results from the PEDV-HM strain, an increase in the EGFP signal was observed in the IFITM1-overexpressing 2D organoids (Fig. 9E). Collectively, these results demonstrate that the overexpression of IFITM1 in porcine small intestinal organoids significantly enhances PEDV infection.

## DISCUSSION

In this study, we employed a genome-wide CRISPR/Cas9 loss-of-function screen to identify critical host factors involved in PEDV infection. Our findings establish IFITM3 as a critical entry factor of PEDV infection in human cells. The knockout studies in Huh7 cells demonstrated a significant reduction in PEDV entry and subsequent virion production, underscoring the role of IFITM3 in viral replication. Notably, reintroducing IFITM3 into knockout cells only partially restored susceptibility to PEDV, suggesting that the stoichiometry of the IFITM3 protein is key for facilitating the entry of PEDV. IFITMs are expressed in various cell types and are upregulated in response to type I and type II interferons, which are produced during viral infections (47). The three best-characterized members of the IFITM family are IFITM1, IFITM2, and IFITM3. IFITM1 is localized mainly at the plasma membrane, whereas IFITM2 and IFITM3 are found inside the cell on endolysosomal membranes (48). IFITMs have been shown to restrict the replication of a wide range of enveloped viruses, including influenza A virus, dengue virus, West Nile virus, human immunodeficiency virus, and coronavirus, by modulating the cholesterol levels of endosomal membranes to change their composition, fluidity, and curvature or via cholesterol-independent mechanisms (48–56). However, recent findings suggest that IFITMs can also facilitate the infection of certain viruses. Several studies have demonstrated that IFITM proteins can interact with viral glycoproteins, thereby facilitating the viral entry process. For example, IFITM3 and IFITM2 have been shown to enhance the entry of human coronavirus OC43 by promoting the interaction between the viral spike protein and the host cell receptor (44). Endogenous IFITMs, including IFITM1, IFITM2, and IFITM3, reportedly increase the infectivity of SARS-CoV-2 infection (57). Similarly, IFITMs promote infection by the Nipah virus (58), human cytomegalovirus (59), and hepatitis B and D viruses (60). Our findings extend this concept to PEDV, demonstrating that IFITMs are host factors facilitating PEDV entry and infection. These findings suggest that the role of IFITMs in viral infections is more complex than initially thought and may depend on the specific virus and cellular context.

We also showed that low-dose IFN-β treatment significantly enhanced the entry and replication of PEDV. However, it is important to note that as the dose of IFN-β increases, the increase in viral entry and replication gradually diminishes, indicating a complex relationship between the IFN-β dosage and its efficacy in viral infection. There are several possible explanations for this phenomenon: (i) the stoichiometry of IFITM is essential for the entry of PEDV into host cells and its subsequent replication. Specifically, the level of IFITM required to promote viral replication effectively must be maintained within a specific range to maximize its effects and avoid triggering adverse responses. (ii) IFN-β stimulation can also induce the expression of other interferon-stimulated genes that exhibit significant antiviral effects against PEDV. The expression of these genes can counteract the role of IFITM in facilitating PEDV entry, underscoring the importance of understanding the broader network of antiviral responses triggered by IFN-β, which could complicate its role in viral replication. Understanding this nonlinear response is essential for optimizing treatments and developing effective intervention strategies.

PEDV utilizes various endocytic pathways after attachment, such as clathrin-mediated endocytosis, caveolae-mediated endocytosis, clathrin- and caveolae-mediated endocytosis, and lipid raft-mediated endocytosis. PEDV membrane fusion primarily occurs in late endosomes and lysosomes and relies on a low pH environment and proteolytic cleavage to release viral genomes into the cytoplasm (61, 62). In our studies, we observed that, compared with IFITM3-WT cells, IFITM3-KO Huh7 cells presented a delay in viral RNA amplification, with a difference of at least 3 h. This delay can be attributed to the modulation of endosomal trafficking, as IFITM3 is localized to endosomal compartments and can influence the endocytic pathway. Consequently, alterations in endosomal trafficking and maturation may create a more favorable environment for viral uncoating and early-stage replication.

Our study further elucidates the involvement of IFITM family members in PEDV entry, with a particular emphasis on the divergence between human and porcine IFITM proteins. Porcine IFITM1 significantly increased PEDV infectivity in LLC-PK1 cells, with further increases observed in porcine small intestinal organoids. However, there was no effect of porcine IFITM2/3 on PEDV infection. The differential effects of IFITM family members on PEDV infection suggest a nuanced role in which structural variances, especially at the C-terminus, as demonstrated by porcine IFITM2/3 truncations, dictate the extent of viral facilitation. This truncation study raises intriguing questions regarding the structural domains of IFITM proteins that modulate their interactions with host and viral membranes. As noted in prior work (44), the C-terminal domains of IFITM proteins critically influence their subcellular localization, which in turn may dictate their functional roles during viral entry. The truncation or modification of these C-terminal regions could plausibly alter IFITM trafficking to distinct organelles (e.g., endosomes, lysosomes, or the plasma membrane), potentially reshaping their interactions with PEDV entry machinery. Such relocalization might explain the observed proviral effects, as compartment-specific interactions could either enhance viral fusion or shield virions from degradation. Additionally, IFITM proteins feature a transmembrane topology (Fig. S7) that enables the C-terminal ectodomain to serve as a binding interface for the S1 component of PEDV virions. This ectodomain likely facilitates viral attachment or fusion, as evidenced by the impairment of PEDV entry when C-terminal truncations occur. Therefore, both the localization and structure of IFITM proteins are integral to their function in viral entry. Our experimental results, as depicted in Fig. 7A, demonstrated that the removal of the 15 C-terminal amino acids from porcine IFITM2/3 resulted in increased PEDV entry. The intricate interplay between the C-terminal regions of IFITMs and viral entry mechanisms highlights the importance of these molecular interactions in the context of viral infections. The detailed molecular mechanisms by which IFITM proteins promote PEDV entry remain to be further explored.

Our study has several limitations that warrant consideration. First, while our data strongly support the role of IFITM3 in promoting PEDV entry and replication, we cannot fully exclude the potential contribution of IFITM2, a homolog with high sequence similarity to IFITM3. Residual IFITM2 expression in IFITM3-KO Huh7 cells, as suggested by Western blot analysis (Fig. S1C), may partially compensate for the loss of IFITM3. This possibility is further supported by the observation that overexpression of IFITM2 in Huh7.5 cells (which lack endogenous IFITM2) significantly enhances PEDV entry (Fig. 3C through E). The functional redundancy between IFITM2 and IFITM3, coupled with their overlapping induction by IFN, complicates the precise delineation of their individual roles in PEDV infection. Second, the incomplete restoration of PEDV susceptibility in IFITM3-reconstituted KO cells (Fig. 3) suggests that IFITM3 expression levels or additional host factors may fine-tune viral entry efficiency.

In conclusion, this study identified IFITMs as significant entry factors for PEDV. This finding is important because it elucidates a fundamental aspect of the molecular interactions between PEDV and its host cells, highlighting the potential of IFITMs as therapeutic targets for managing PEDV infections, paving the way for novel antiviral strategies. Furthermore, the broader implications of this research underscore the crucial role of host factors in controlling the spread of pathogenic coronaviruses. Specifically, the findings suggest that manipulating the domains of IFITMs could be essential for effectively managing infectious diseases that involve direct transmission from livestock to humans.

## MATERIALS AND METHODS

### Plasmid construction

Small guide RNA sequences targeting human IFITM3 were designed (https://www.zlab.bio/resources), synthesized, cloned, and inserted into the lentiCRISPRv2-puro plasmid via T4 DNA ligase (NEB, Beijing, China). The lentiviral vectors pLV-Puro-U6-Scramble-shRNA, pLV-Puro-U6-hIFITM3-shRNA1, and pLV-Puro-U6-hIFITM3-shRNA2 were obtained from VectorBuilder (Guangzhou, China). A human codon-optimized, full-length S gene for the PEDV SD strain (GenBank accession no. MZ596343) was synthesized by Saiheng Biotech (Shanghai, China) and inserted into the pLV-EF1a-IRES-Hygro (Addgene #85134) vector to generate pLV-EF1a-PEDV-S-IRES-Hygro. A human codon-optimized S1 gene (832 aa) with an Fc tag for the PEDV SD strain was also synthesized by Saiheng Biotech (Shanghai, China) and inserted into the pcDNA3.1(+) vector to generate pcDNA3.1-PEDV-S1-Fc. The expression vectors for the SARS-CoV-2 spike protein and MERS-CoV spike protein in pcDNA3.1(+) were generously provided by Prof. Rong Zhang (Fudan University). The human IFITM1, IFITM2, IFITM3, porcine IFITM1, and IFITM2/3 genes were synthesized by Saiheng Biotech (Shanghai, China) and cloned and inserted into pLV-EF1α-IRES-Hygro to generate pLV-EF1α-Human-IFITM1-IRES-Hygro, pLV-EF1α-Human-IFITM2-IRES-Hygro, pLV-EF1α-Human-IFITM3-IRES-Hygro, pLV-EF1α-Porcine-IFITM1-IRES-Hygro, and pLV-EF1α-Porcine-IFITM2/3-IRES-Hygro vectors. All of the IFITM genes with the HA tag were also cloned and inserted into pLV-EF1α-IRES-Hygro via homologous recombination. The primers used in this study are listed in Table S1.

### Cells, viruses, and antibodies

Vero CCL-81 (ATCC), Huh7 (a gift from Prof. Rong Zhang, Fudan University), Huh7.5 (a gift from Prof. Rong Zhang, Fudan University), HEK293T (ATCC, CRL-3216), and LLC-PK1 (MeiSenCTCC, Zhejiang, China) cells were cultured in Dulbecco's modified Eagle medium (DMEM) (HyClone, Shanghai, China) containing 10% fetal bovine serum (FBS) (Gibco, Shanghai, China). The PEDV SD strain (GenBank accession no. MZ596343) and PEDV-HM strain (GenBank accession no. MZ342899) were stored in our laboratory. The recombinant virus rPEDV-HM-EGFP was produced by transfecting infectious cDNA clones into BHK21 cells, as previously described (46). The recombinant virus rVSV-∆G-EGFP-G was obtained from VectorBuilder (VB010000-9315gcp, Guangzhou, China). The PEDV pseudovirus rVSV-∆G-EGFP-PEDV-S was generated by infecting Huh7 cells overexpressing the PEDV spike protein with rVSV-∆G-EGFP-G. The pseudoviruses rVSV-∆G-EGFP-SARS-CoV-2-S and rVSV-∆G-EGFP-MERS-CoV-S were produced by infecting HEK293T cells transfected with the expression vectors of SARS-CoV-2 spike and MERS-CoV spike with rVSV-∆G-EGFP-G, respectively. A monoclonal antibody for PEDV N (cat#: YY151229) was purchased from AnChi Biotech (Shanghai, China). A monoclonal antibody for human IFITM1 (cat#: 60074-1-lg) was obtained from Proteintech (Shanghai, China). A polyclonal antibody for human IFITM3 (cat#: 11714-1-AP) was obtained from Proteintech. A monoclonal antibody for β-tubulin (cat#: 66240-1-Ig) was obtained from Proteintech. A monoclonal antibody against GAPDH (cat#: HC301-01) was obtained from TransGen Biotech (Beijing, China). A monoclonal antibody for Flag (cat#: F1804-1MG) was obtained from Sigma (Darmstadt, Germany). A monoclonal antibody for HA (cat#: 3724S) was obtained from Cell Signaling Technology (Danvers, MA, USA). A horseradish peroxidase (HRP)-linked antibody for human IgG, Fc gamma fragment specific (cat#: 32935S), was obtained from Cell Signaling Technology. HRP-linked secondary antibodies for mouse IgG (cat#: SA00001-1) and rabbit IgG (cat#: SA00001-2) were purchased from Proteintech. Goat anti-mouse IgG (H+L) secondary antibody conjugated with Alexa Fluor 594 (cat#: A11032) and Alexa Fluor 488 goat anti-rabbit IgG (H+L) (Cat#: A11008) was purchased from Invitrogen (Shanghai, China). Additional antibodies included EEA1 mouse monoclonal antibody (cat#: 68065-1-Ig), HA tag mouse monoclonal antibody (cat#: 66006-2-Ig), Rab7 (D95F2) XP rabbit monoclonal antibody (cat#: 9367s), and CLTC monoclonal antibody (cat#: 66487-1-Ig) from Proteintech. CD107a/LAMP1 monoclonal antibody (cat#: 67300-1-Ig) was also sourced from Proteintech. The application of each antibody followed the guidelines provided by the respective manufacturer.

### Lentivirus production and concentration

Lentivirus production and concentration were conducted as previously described. Briefly, HEK293T cells were cotransfected with the lentiviral vectors, the packaging plasmid psPAX2 (Addgene, cat#: 12260), and the envelope-coding plasmid pMD2. G (Addgene cat#: 12259) via the calcium phosphate method. At 48 h post-transfection, virus-containing supernatants derived from these HEK293T cultures were clarified by centrifugation at 2,000 rpm for 10 min and filtered through a 0.45 µm cellulose acetate filter. The viral supernatants were then concentrated via PEG6000, aliquoted, and stored at −80°C for future use.

### Lentivirus transduction and cell line establishment

Huh7, Huh7.5, and LLC-PK1 cells were seeded at a density of 2 × 10^5^ per well in six-well plates. Eight hours after seeding, 5 MOIs of viruses supplemented with 8 µg/mL polybrene were used to infect the cells. At 48 hpi, cell culture medium containing 10 µg/mL blasticidin (cat#: ant-bI, InvivoGen), 500 µg/mL hygromycin B (cat#: 10843555001, Roche), or 2.5 µg/mL puromycin (cat#: ant-pr, InvivoGen) was added on the basis of the resistance gene of the lentiviral vector used. The medium was changed every 2–3 days to select for positive cells. For certain experiments, monoclonal cells were isolated through limited dilution in 96-well plates. The expression of the corresponding protein was subsequently verified via Western blotting, and the best overexpressing cell lines were selected for subsequent experiments.

### Genome-wide CRISPR/Cas9 screen

The human GeCKO v2 CRISPR knockout pooled library module B (Addgene #1000000049) containing 58,028 sgRNAs targeting 19,050 genes was a gift from Feng Zhang (63). This library was amplified in Lucigen Endura electrocompetent cells following the author’s recommendations. For lentivirus packaging, HEK293T cells were cotransfected with the lentiviral library and helper plasmids psPAX2 and pMD2.G using calcium phosphate. Viral supernatants were collected at 48 h post-transfection, clarified by centrifugation at 2,000 rpm for 10 min, and filtered through a 0.45 µm cellulose acetate filter. The viral supernatants were subsequently concentrated via PEG6000, aliquoted, and stored at −80°C for further use. For cell transduction, Huh7-Cas9 cells were seeded in 10 T175 flasks at a density of 2 × 10^7^ per flask and infected with 0.3 MOI lentivirus to ensure that each sgRNA had at least 1,000 coverage in the cells. At 48 h post-transduction, puromycin (2.5 µg/mL) was added for 4 days to select successfully transduced cells. The puromycin-resistant cells were then challenged with the PEDV strain SD at an MOI of 0.01 or 0.1. Surviving cells were harvested, and genomic DNA was isolated via the Blood & Cell Culture DNA Midi Kit (Qiagen). The sgRNA sequences present in the collected DNA were amplified via PCR via primers that attach Illumina sequencing recognition sites and barcodes. The PCR product was purified via the Agencourt AMPure XP bead-bound purification kit. The purified PCR product was then sequenced on an Illumina NextSeq platform. The raw FASTQ files were processed by MAGeCK software (64) with default parameters using the sgRNA sequence list for all genes from the GeCKO v2 library. The numbers of uniquely aligned reads for each library sequence were calculated. The numbers of reads for each unique sgRNA for a given sample were subsequently normalized.

### RT-qPCR assay

To quantify the viral genome copy number, viral RNA in the supernatant was extracted via a DNA/RNA extraction kit (Vazyme, Nanjing, China). Reverse transcription was then performed on an equal volume of the RNA eluent using 5× PrimeScript RT Master Mix (Takara, Beijing, China). Subsequently, quantitative real-time PCR was performed in triplicate via 2× ChamQ Universal SYBR qPCR Master Mix (Vazyme, Nanjing, China) on a LightCycler 96 instrument (Roche, Shanghai, China) to detect the coding sequence of the N of PEDV. Absolute quantitative RNA levels were calculated via standard curves generated from the virus stock.

For the detection of IFITM3 mRNA expression, total RNA was isolated from the cells via an RNA Easy Fast Tissue/Cell Kit (cat#: DP451; Tiangen, Shanghai). cDNA was subsequently synthesized via a PrimeScript RT Reagent Kit (cat#: RR047A, Takara) with 1 µg of total RNA. qPCR was carried out via the use of KAPA SYBR Fast Universal qPCR Master Mix (cat#: KK4602, Kapa Biosystems) on a Roche LightCycler96 PCR System instrument. Relative gene expression levels were calculated via the use of GAPDH as the reference gene. The sequences of primers used in these assays can be found in Table S1.

### Virus titration

For PEDV titration, Vero cells were cultured in 96-well plates to 100% confluence. The viral samples were diluted in a 10-fold gradient, and each dilution was inoculated in eight replicates in 100 µL. Each sample was repeated in three groups. The cells were then cultured for another 5 days and observed for cytopathic lesions. The virus titers were calculated via the Reed-Muench method and expressed as TCID_50_/mL.

### Packaging of rVSV-∆G-EGFP-SARS-CoV-2-S and rVSV-∆G-EGFP-MERS-CoV-S pseudoviruses

To generate rVSV-∆G-EGFP-SARS-CoV-2-S and rVSV-∆G-EGFP-MERS-CoV-S pseudoviruses, approximately 3 × 10^6^ HEK293T cells were seeded in a T75 flask. After 24 h, the cells were transfected with 24 µg of either pCAGGS-SARS-CoV-2-S or MERS-CoV-S via the calcium phosphate method. The transfected cells were incubated for 24 h at 37°C with 5% CO_2_. The cells were then infected with 2 MOIs of rVSV-∆G-EGFP-G to produce pseudoviruses. After 2 h, the viral medium was removed, and the cells were thoroughly washed five times with Dulbecco's phosphate-buffered saline (DPBS) to remove any residual virus. Then, 15 mL of DMEM containing 2% FBS was added to sustain cell growth and viral production. The supernatants containing the pseudoviruses were collected at 48 h hpi, centrifuged to remove cell debris, filtered for purity, aliquoted, and stored at −80°C.

### Packaging of the rVSV-∆G-EGFP-PEDV-S pseudovirus

To produce the rVSV-∆G-EGFP-PEDV-S pseudovirus, a monoclonal cell line that highly expresses the spike protein of the PEDV SD strain was infected with an rVSV-∆G-EGFP-G pseudovirus at an MOI of 2. Following a 2 h incubation period, the virus was discarded, and the cells were subsequently washed three times with phosphate-buffered saline (PBS) and cultured in fresh DMEM supplemented with 2% FBS. The supernatant was collected at 48 hpi, centrifuged, filtered for purity, aliquoted, and stored at −80°C.

### IFITM3-knockout in Huh7 and LLC-PK1 cells

IFITM3-KO Huh7 cells were generated as previously described (45, 65, 66). Briefly, Huh7 cells were transfected with a pair of small guide RNAs in the lentiCRISPRv2-puro (cat#: 98290, Addgene) vector via Lipofectamine 3000 Transfection Kits (cat#: L3000-015, Invitrogen). After 24 h, fresh medium supplemented with 2.5 µg/mL puromycin was added, and the cells were incubated for 3 days. Single-positive cells were then manually diluted in 96-well plates. Single-cell clones were expanded and validated via Western blotting and DNA sequencing.

### IFITM3-knockdown in Huh7 cells

To achieve knockdown of the IFITM3 gene, Huh7 cells were transduced with lentiviruses encoding the shRNAs targeting human IFITM3 described in Table S1. The cells were then incubated for 12 h at 37°C, washed with PBS, and added to a fresh medium. At 96 h post-transfection, a medium containing 2.5 µg/mL puromycin was added for 3 days. The knockdown efficiency was subsequently confirmed via quantitative PCR and Western blot analysis to measure the reduction in IFITM3 mRNA and protein levels, respectively.

### Flow cytometry analysis

Flow cytometry was used to determine the percentage of cells infected by the pseudotyped viruses. In brief, virus-infected cells were rinsed with DPBS, treated with trypsin/EDTA, and suspended in 1 mL of fluorescence-activated cell sorting buffer (PBS containing 2 mM EDTA, 0.1% sodium azide, and 2% FBS). The number of GFP-positive cells was determined via a CytoFLX flow cytometer (Beckman Coulter). The acquisition was set for 50,000 events per sample. The data were analyzed via FlowJo software. All the samples were prepared in triplicate.

### Virus attachment and internalization assay

For viral attachment, PEDV-infected Huh7 cells (MOI = 10) were incubated on ice for 1 h and then washed with cold PBS three times, and viral attachment to the cell surface was detected by RT-qPCR. For viral internalization, virus-attached cells were incubated at 37°C for another 1 h, and viral internalization was detected by RT-qPCR. For viral entry, Huh-7 cells were infected with PEDV (MOI = 1) at 37°C for 6 h. The cells were then harvested, and RNA copies were detected via RT-qPCR.

### Western blotting

Cell lysates were prepared via radioimmunoprecipitation assay (RIPA) buffer (cat#: 89900, Thermo Scientific, Waltham, MA, USA) supplemented with a protease inhibitor cocktail (cat#: ST506-2, Beyotime). The cell lysates mixed with loading buffer were boiled at 100°C for 10 min and separated by electrophoresis on a 7.5% or 10% SDS-PAGE gel. The blot was blocked with TBST (10 mM Tris-HCl, pH 7.5, 150 mM NaCl, and 0.1% Tween-20) containing 5% nonfat dry milk and then incubated with the primary antibody in western primary antibody diluent at 4°C overnight. After being washed three times with TBST, the membrane was incubated with HRP-conjugated secondary antibody for 1 h at room temperature. The signals were visualized with an NcmECL Ultra chemiluminescence kit (cat#: P10200, NCM Biotech, Suzhou, China) and detected via a ChemiDocMP imaging system (Bio-Rad, Hercules, CA, USA).

### Co-IP

HEK293T cells were seeded in a T75 flask. Upon reaching 80% confluence, the cells were cotransfected with the specified plasmids via Lipofectamine 3000 Transfection Kits (Invitrogen, Shanghai, China). Eight hours after transfection, the medium was replaced with fresh media containing 10% FBS. Twenty-four hours after transfection, the cells were lysed with IP lysis buffer (Thermo Fisher Scientific, Shanghai, China) for 15 min on ice, followed by centrifugation to collect the supernatant. Approximately 10% of the lysate supernatant was reserved as an input control, while the remaining lysate was incubated with Protein A/G agarose beads (Sigma, Shanghai, China) at 4°C overnight with rotation. The beads were subsequently washed five times with IP lysis buffer, and the proteins were eluted in elution buffer (Thermo Fisher Scientific, Shanghai, China). The eluted proteins were analyzed by SDS-PAGE followed by Western blotting to detect interacting proteins.

### Indirect immunofluorescence assay

The cells were fixed with 80% cold ethanol at 4°C for 30 min, washed three times with PBS, and blocked with 5% bovine serum albumin (BSA) at 37°C for 1 h. Then, the cells were incubated with primary antibody for 1.5 h and washed with PBS three times. Next, the cells were incubated with a goat anti-mouse IgG (H+L) antibody conjugated with Alexa Fluor 594 for 1 h and washed with PBS three times. Finally, the cells were counterstained with 5 µg/mL 4′,6′-diamidino-2-phenylindole (DAPI) (Sigma, Shanghai, China) for 10 min. Images were taken with a microscope equipped with a monochrome EMCCD camera (Zeiss, Germany).

### Confocal imaging

Sterile coverslips were placed in a six-well plate, and LLC-PK1-vector and LLC-PK1-HA-pIFITM1 cells were added. When the cells reached 60% confluence, they were fixed in 4% paraformaldehyde for 30 min, blocked with 10% bovine serum albumin for 1 h, and permeabilized with 0.1% Triton X-100 for 15 min. The cells were then incubated with primary antibodies for 2 h at 37°C, followed by three washes with PBS. The cells were subsequently incubated with secondary antibodies at 37°C for 1 h, stained with 1 µg/mL DAPI for 10 min, and examined via a Zeiss confocal microscope.

To evaluate the colocalization of the S protein and porcine IFITM1 during PEDV infection, LLC-PK1-vector and LLC-PK1-HA-pIFITM1 cells were subsequently infected with the PEDV-HM strain at an MOI of 0.1 for 6 h. After infection, the cells were washed three times with PBS to remove any unbound virus. At 24 hpi, the colocalization of the S protein and IFITM1 was assessed through confocal imaging, as previously described, using anti-S protein and anti-HA antibodies.

### Porcine intestinal organoid culture and transduction

Porcine intestinal organoids derived from 10-day-old pig intestines were cultured as previously described (43). Briefly, intestinal crypts from the small intestine were isolated, subsequently resuspended in Matrigel, and plated in 50 µL droplets in each well of a 24-well plate. After allowing the Matrigel to solidify, an organoid culture medium supplemented with 10 µM ROCK inhibitor was added to the plates, and the organoids were cultured at 37°C with 5% CO_2_.

To achieve overexpression of porcine HA-IFITM1 in porcine small intestinal organoids, the organoids were collected in cold advanced DMEM/F12 (ADF) and washed once before being digested with TrypLE (Invitrogen, Shanghai, China). After digestion, the organoids were washed once with an ADF medium. The organoids were then infected with lentivirus expressing porcine HA-IFITM1 or with an empty vector as a control at an MOI of 5. After incubation for 2 h, the cultures were washed twice with ADF medium to remove unbound viruses. The organoids were reembedded in Matrigel in 24-well plates and cultured in an organoid culture medium. At 48 hpi, the organoids were cultured with 2.5 µg/mL puromycin for 2 weeks. The organoids that overexpressed either HA-IFITM or the empty vector were expanded and subsequently used for PEDV infection.

### Culture of 2D porcine intestinal organoids and PEDV infection

To culture 2D porcine intestinal organoids, three-dimensional porcine intestinal organoids were initially digested with TrypLE at 37°C for 10 min. Subsequently, the cell suspension was filtered through a 40 µm filter and centrifuged to remove the supernatant. The cells were resuspended in advanced DMEM/F12 medium supplemented with 10% L-WRN conditional medium, 10% FBS, 5 µM DAPT {N-[N-(3,5-difluorophenacetyl)-L-alanyl]-S-phenylglycine t-butyl ester} (γ-secretase inhibitor), and 10 µM ROCK inhibitor and plated into a 12-well plate coated with 1% Matrigel.

Once the 2D porcine intestinal organoids reached confluence, they were infected with either PEDV-HM or rPEDV-HM-EGFP at an MOI of 0.1 for 1 h. At 24 hpi, the supernatant containing the PEDV-HM was collected, and the copy number of the PEDV genome was quantified via RT-qPCR. Additionally, the infection efficiency of rPEDV-HM-EGFP was assessed via fluorescence microscopy.

### Statistical analysis

The data analysis was conducted via GraphPad Prism 9 (GraphPad, San Diego, CA, USA). The data are expressed as the mean ± standard deviation of at least three replicates. Unpaired Student’s *t*-tests were performed to calculate the *P*-values. The significance level (*P*-value) was set at <0.05 (*), <0.01 (**), <0.001 (***), and <0.001 (****).

## Data Availability

All data generated or analyzed during this study are included in this article and its supplemental material. All other raw data supporting the conclusions of this study are available from the corresponding author upon reasonable request.

## SUPPLEMENTAL MATERIAL

Fig. S1 jvi.02028-24-s0001.tif The genome-wide CRISPR/Cas9 screen identified host factors critical for PEDV infection.

Fig. S2 jvi.02028-24-s0002.tif Endogenous IFITM3 expression enhances PEDV infection.

Fig. S3 jvi.02028-24-s0003.tif Effects of human IFITM1 on PEDV infection.

Fig. S4 jvi.02028-24-s0004.tif IFITM3 facilitates PEDV entry into host cells.

Fig. S5 jvi.02028-24-s0005.tif Sequence alignment of the human and porcine IFITM1, IFITM2, and IFITM3 proteins.

Fig. S6 jvi.02028-24-s0006.tif Interaction of the PEDV S1 protein with human IFITM3 and porcine IFITM1.

Fig. S7 jvi.02028-24-s0007.tif IFITM membrane topology models illustrating different proposed locations of the N-terminal domain (NTD), C-terminal domain (CTD), and conserved intracellular loop (CIL).

Supplemental legends jvi.02028-24-s0008.docx Legends for Fig. S1 to S7.

Table S1 jvi.02028-24-s0009.docx Oligo sequences used in this study.

ASM does not own the copyrights to Supplemental Material that may be linked to, or accessed through, an article. The authors have granted ASM a non-exclusive, world-wide license to publish the Supplemental Material files. Please contact the corresponding author directly for reuse.
