# Supplementary figures and images for: IFITM proteins are key entry factors for porcine epidemic diarrhea coronavirus
Source: J Virol. 2025 May 12;99(6):e02028-24. doi: 10.1128/jvi.02028-24 (PMC12172462; doi:10.1128/jvi.02028-24)

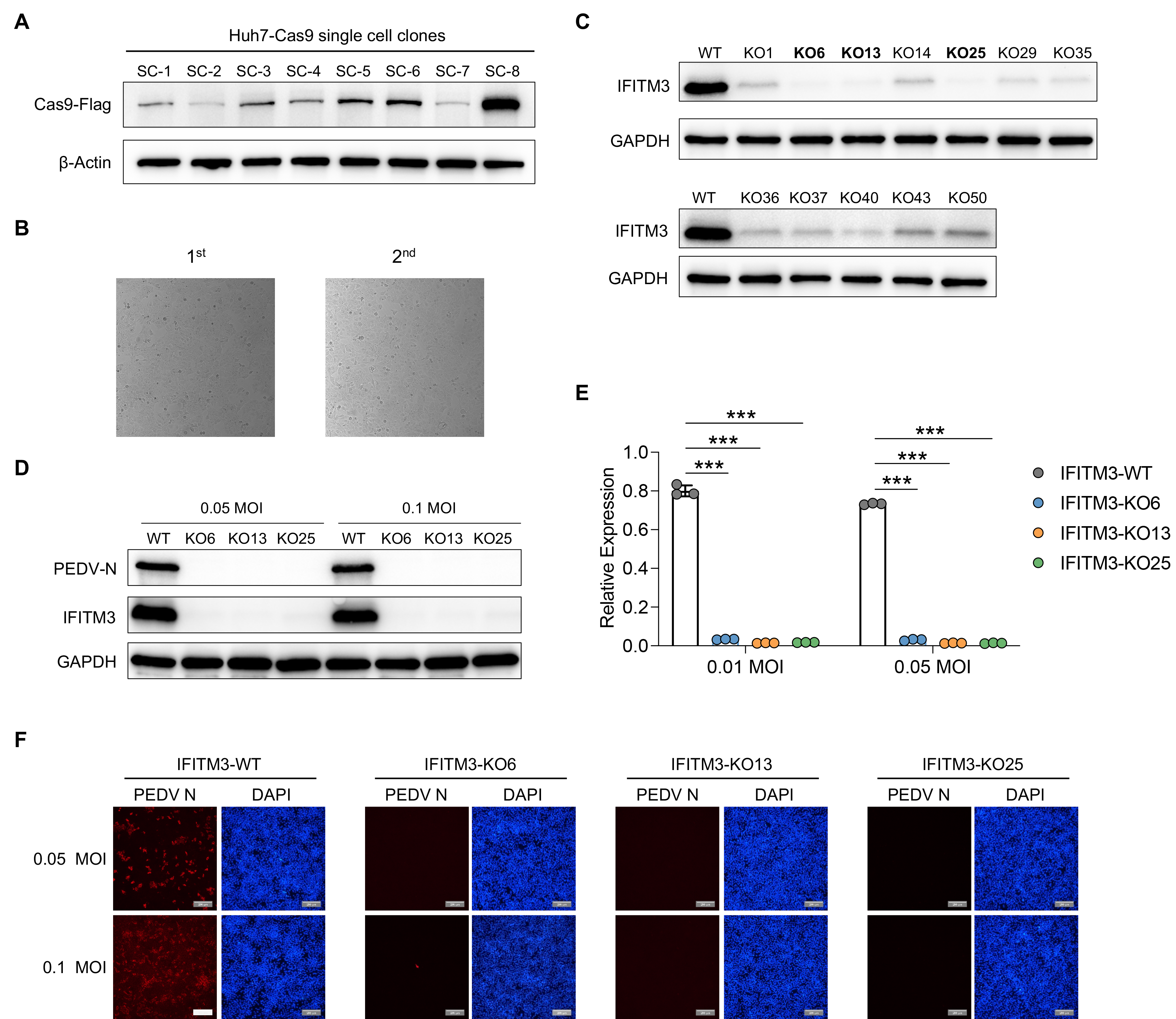

Supplement: Fig. S1 — The genome-wide CRISPR/Cas9 screen identified host factors critical for PEDV infection. [file jvi.02028-24-s0001.tif]

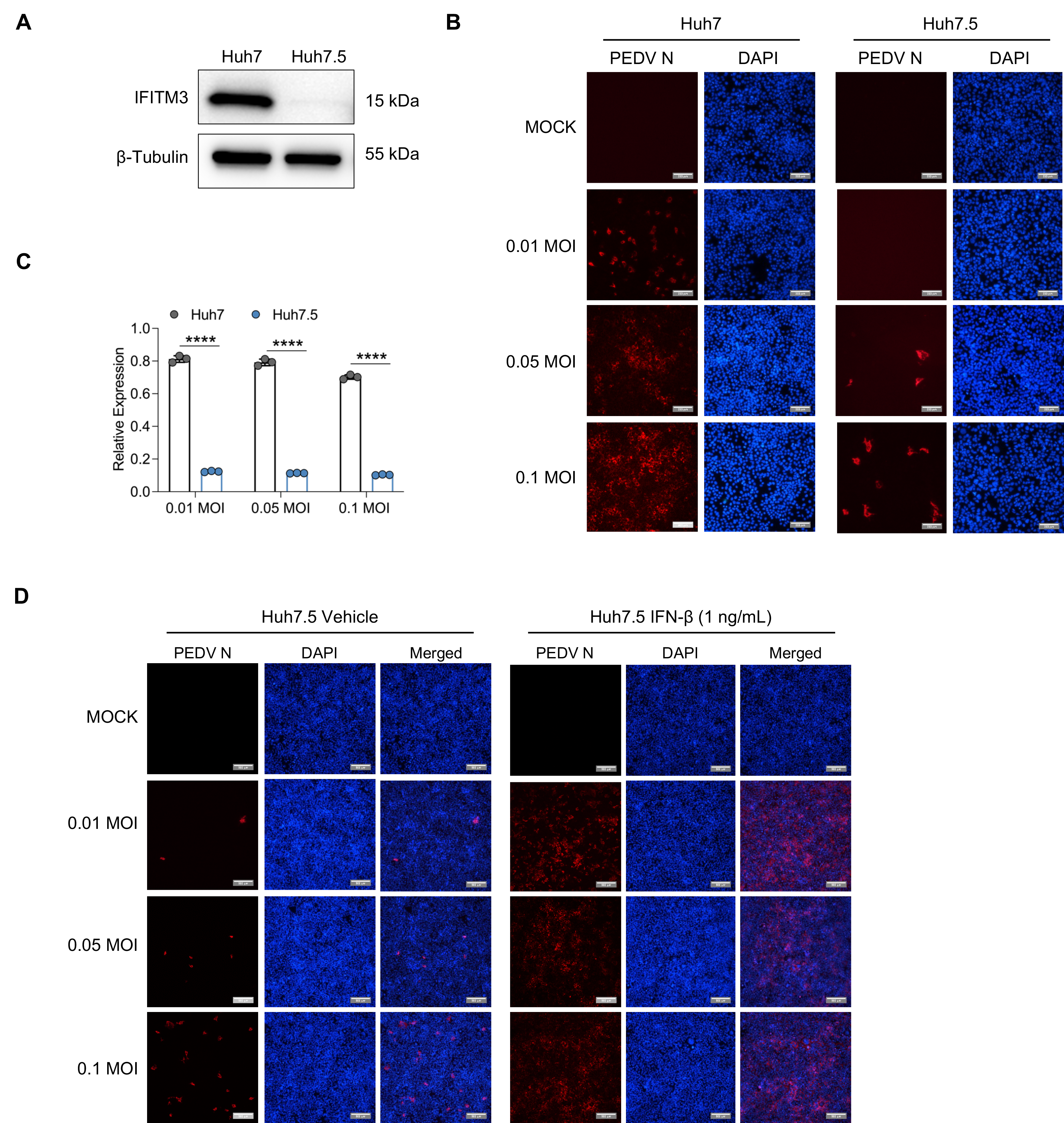

Supplement: Fig. S2 — Endogenous IFITM3 expression enhances PEDV infection. [file jvi.02028-24-s0002.tif]

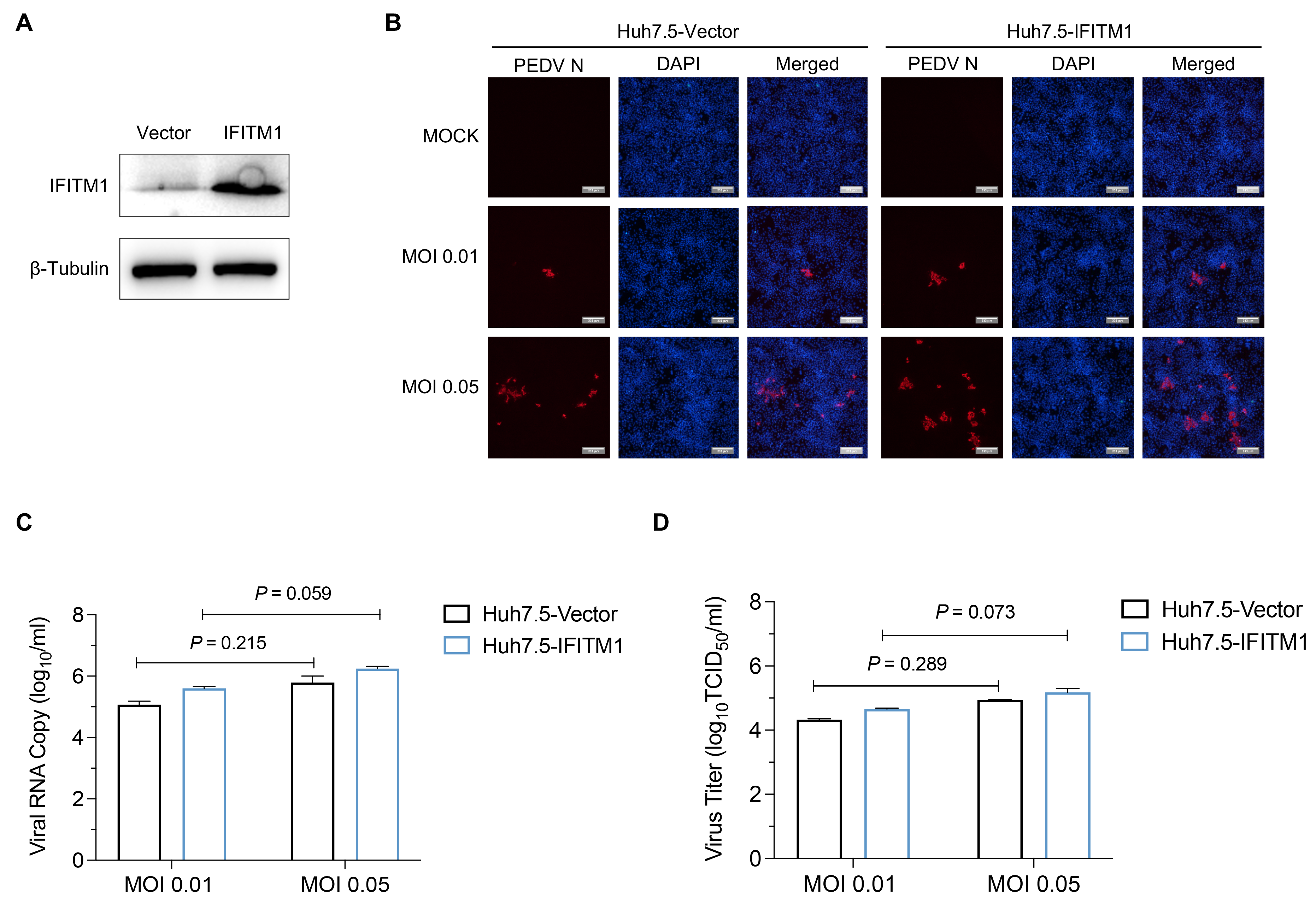

Supplement: Fig. S3 — Effects of human IFITM1 on PEDV infection. [file jvi.02028-24-s0003.tif]

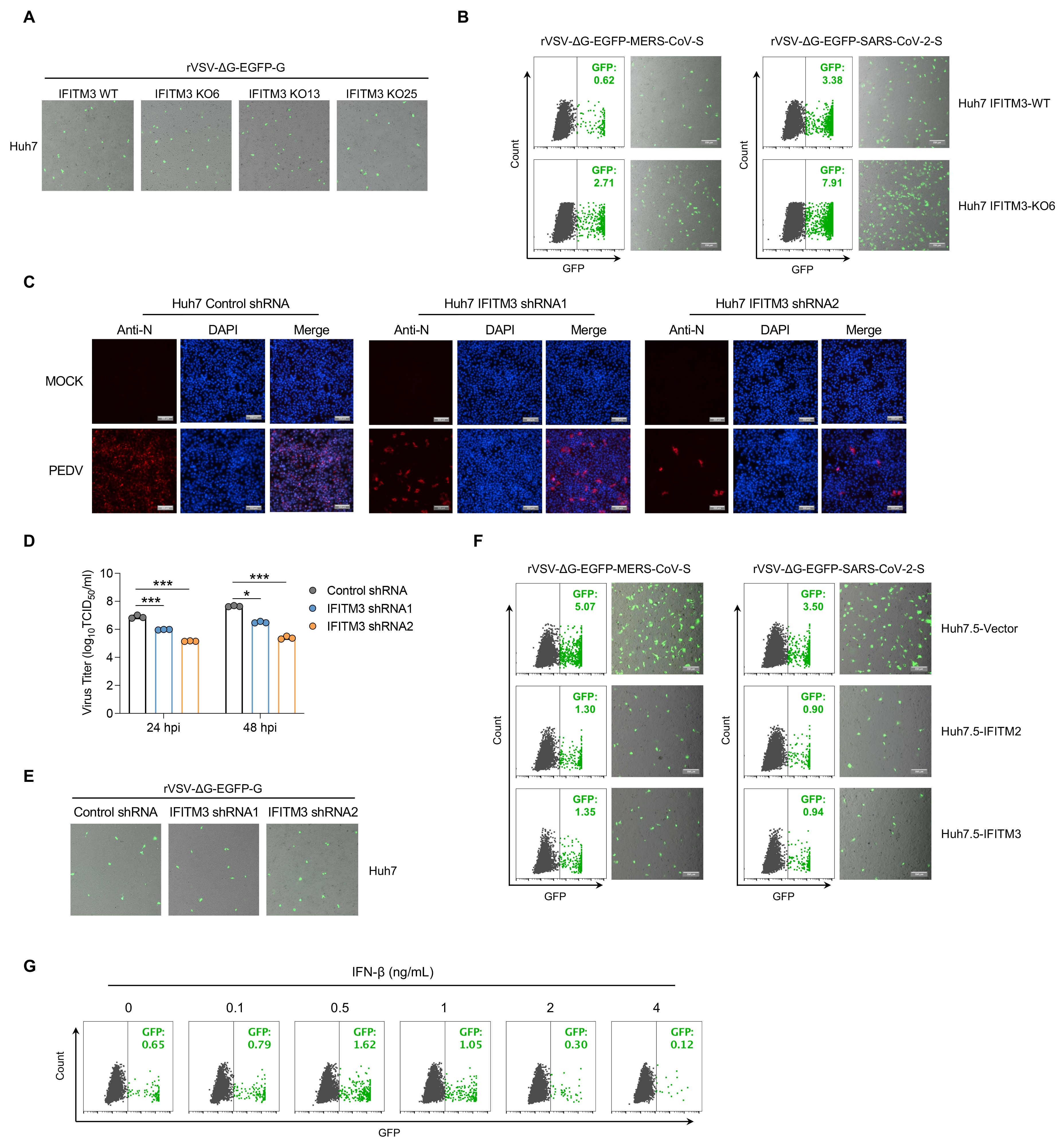

Supplement: Fig. S4 — IFITM3 facilitates PEDV entry into host cells. [file jvi.02028-24-s0004.tif]

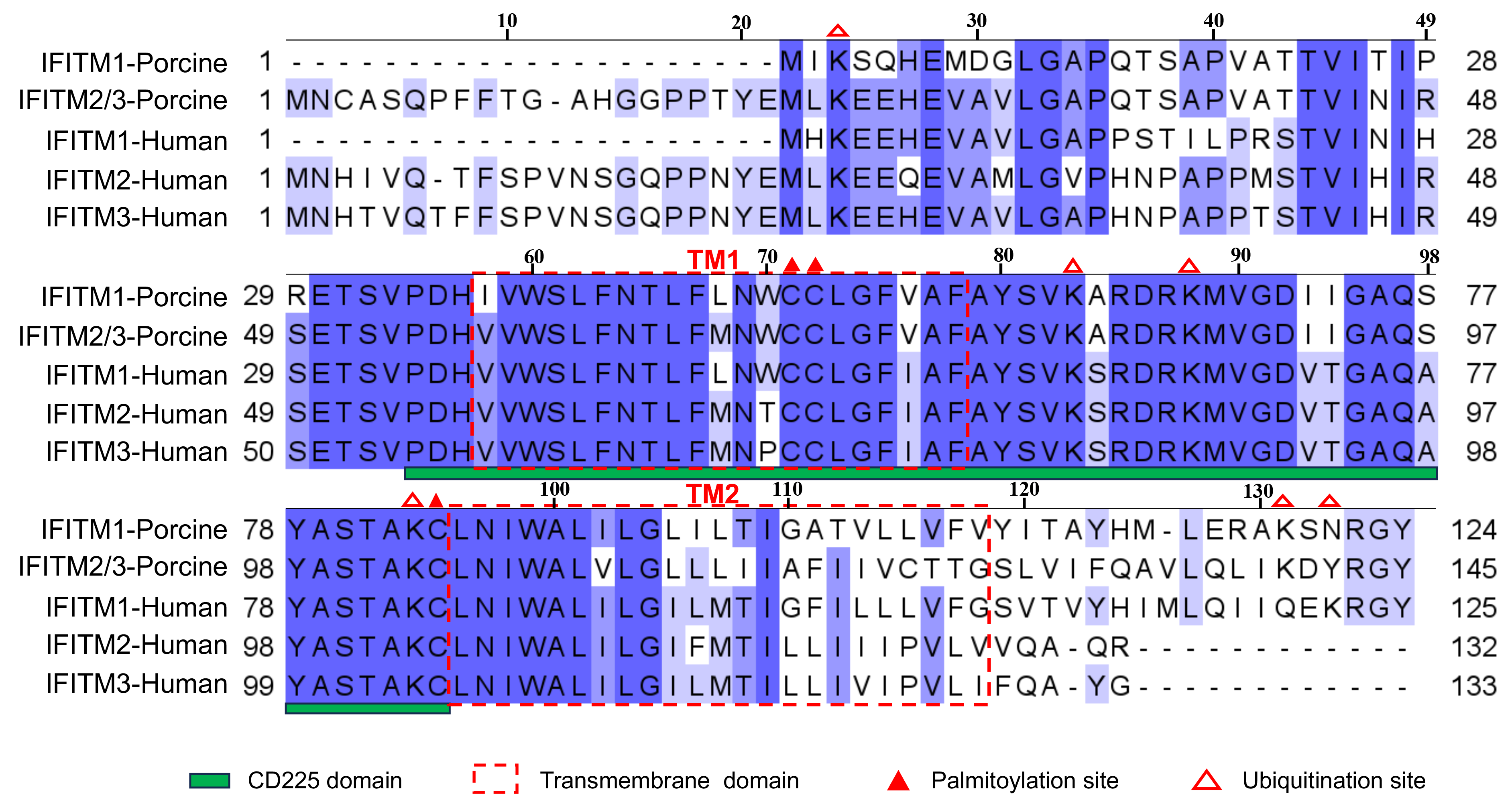

Supplement: Fig. S5 — Sequence alignment of the human and porcine IFITM1, IFITM2, and IFITM3 proteins. [file jvi.02028-24-s0005.tif]

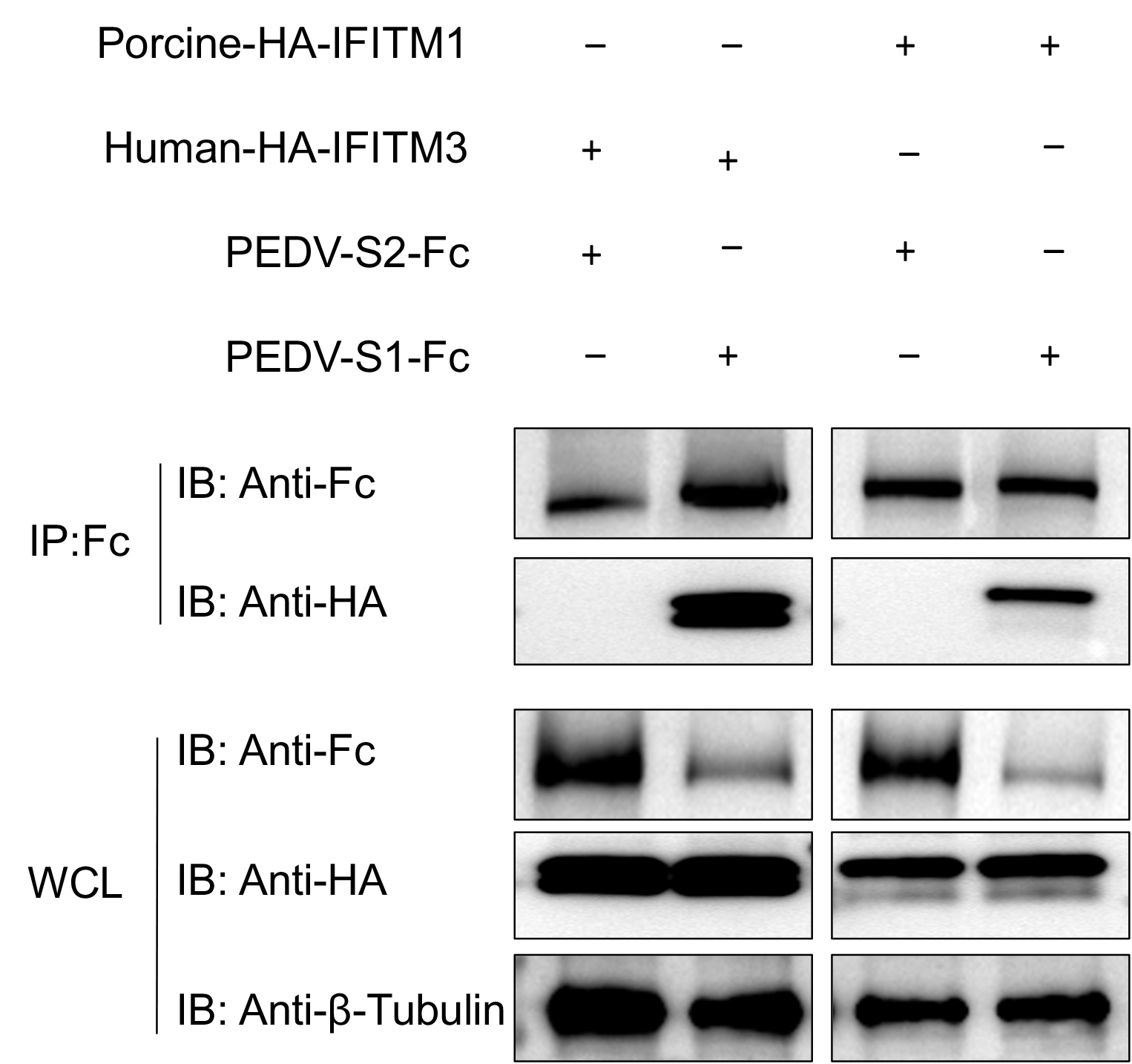

Supplement: Fig. S6 — Interaction of the PEDV S1 protein with human IFITM3 and porcine IFITM1. [file jvi.02028-24-s0006.tif]

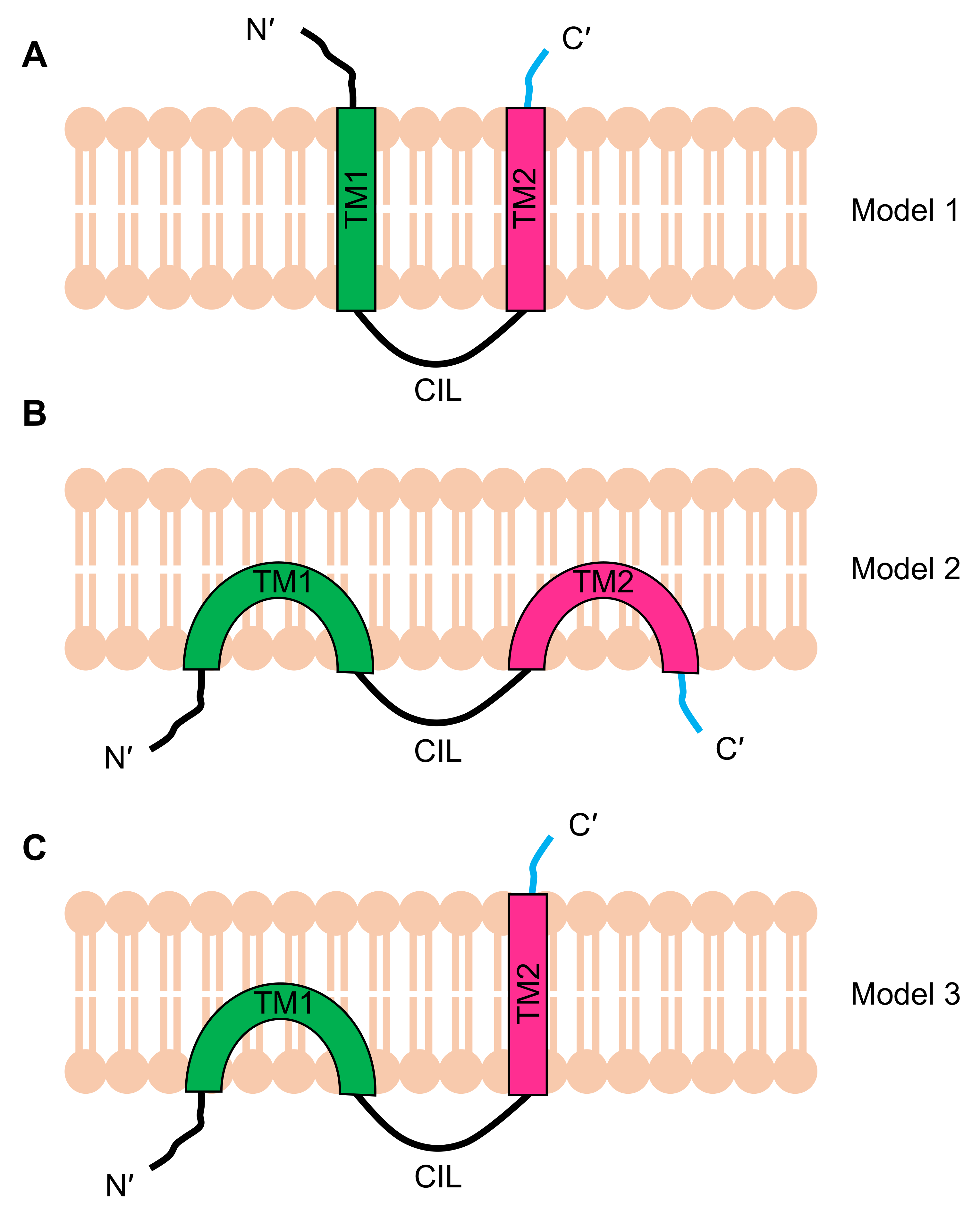

Supplement: Fig. S7 — IFITM membrane topology models illustrating different proposed locations of the N-terminal domain (NTD), C-terminal domain (CTD), and conserved intracellular loop (CIL). [file jvi.02028-24-s0007.tif]
