# Supplementary material for: IFITM proteins are key entry factors for porcine epidemic diarrhea coronavirus
Source: J Virol. 2025 May 12;99(6):e02028-24. doi: 10.1128/jvi.02028-24 (PMC12172462; doi:10.1128/jvi.02028-24)
Supplement: Supplemental legends — Legends for Fig. S1 to S7. [file jvi.02028-24-s0008.docx]

**Figure S1. The genome-wide CRISPR/Cas9 screen identified host factors critical for PEDV infection**. (A) Western blot analysis shows the stable expression of the Cas9 protein in different single-cell clones of Huh7 cells. Among these clones, SC-8 presented the highest expression of Cas9 and was selected for genome-wide library screening. (B) The morphology of Huh7-Cas9 cells from two independent screens is presented. (C) Western blot analysis shows the expression of IFITM3 in various single-cell clones derived from pooled IFITM3-knockout Huh7 cells. IFITM3 knockout clones KO6, KO13, and KO25 were selected for further use. (D) Western blot analysis illustrating the expression of the PEDV N protein and IFITM3 protein in IFITM3 knockout Huh7 cells infected with different MOIs (0.05 and 0.1) of PEDV at 12 hpi. (E) The grayscale values of the N protein bands in Figure S1D were quantitatively analyzed with the assistance of ImageJ software. The error bars indicate the s.d.s from three technical repeat. ***, *P* < 0.001. (F) Representative immunofluorescence images of the PEDV N protein corresponding to Figure 1D are presented. Scale bar: 200 μm.

**Figure S2. Endogenous IFITM3 expression enhances PEDV infection.** (A) Western blot demonstrating the expression of IFITM3 in Huh7 and Huh7.5 cells. β-Tubulin served as a loading control. (B) Representative immunofluorescence images of the PEDV N protein related to Figure 2A. Red indicates the viral N protein, and blue denotes the cell nuclei (DAPI). Scale bar: 200 μm. (C) The grayscale values of the N protein bands in Figure 2B were quantitatively analyzed with the assistance of ImageJ software. The error bars indicate the s.d.s from three technical repeat. ****, *P* < 0.0001. (D) Huh7.5 cells were treated with 1 ng/ml IFN-β for 12 h. Subsequently, the cells were infected with PEDV at MOIs of 0.01, 0.05 and 0.1. At 12 hpi, the cells were fixed and visualized via immunofluorescence staining. Representative fluorescence images showing the intracellular PEDV N protein in the cells. Red indicates the viral N protein, and blue denotes the cell nuclei (DAPI). Scale bar: 200 μm.

**Figure S3. Effects of human IFITM1 on PEDV infection.** (A) Huh7.5 cells were transduced with lentiviral vectors expressing human IFITM1 proteins or an empty vector as a control. Western blot showing the overexpression of human IFITM1 in Huh7.5 cells. β-Tubulin served as a loading control. (B) IFITM1-overexpressing Huh7.5 cells and control cells were infected with PEDV at MOIs of 0.01 and 0.05, respectively. At 12 hpi, the cells were fixed and visualized via immunofluorescence staining. Representative fluorescence images showing the intracellular PEDV N protein in the cells. Red indicates the viral N protein, and blue denotes the cell nuclei (DAPI). Scale bar: 200 μm. (C) The viral RNA in the supernatants was quantified by qRT-PCR and is presented as the viral RNA copy number per millilitre. The error bars indicate the s.d. from three biological replicates (n = 3). (F) Infectious PEDV particles in the supernatants of these cells were assessed via the TCID_50_ assay. The error bars indicate the s.d. from three independent experiments. ***, *P* < 0.001.

**Figure S4. IFITM3 facilitates PEDV entry into host cells.** (A) IFITM3-WT and IFITM3-KO Huh7 cells were infected with equal amounts of the rVSV-ΔG-EGFP-G virus. The samples were analyzed via fluorescence microscopy at 12 hpi. (B) IFITM3 WT and IFITM3 KO Huh7 cells were infected with equal amounts of rVSV-ΔG-EGFP-MERS-CoV-S or rVSV-ΔG-EGFP-SARS-CoV-2-S. Samples were analyzed via fluorescence microscopy or flow cytometry. (C) Huh7 cells were transduced with lentiviral vectors containing either IFITM3 shRNAs or scramble shRNA and infected with PEDV. At 12 hpi, the cells were fixed and stained for the PEDV N protein. Representative immunofluorescence images are shown. Red indicates the viral N protein, and blue denotes the cell nucleus (DAPI). Scale bar: 200 μm. (D) Huh7 cells were transduced with lentiviral vectors containing either IFITM3 shRNAs or scramble shRNA and infected with PEDV. At 24 and 48 hpi, the number of infectious PEDV particles in the supernatants was assessed via the TCID_50_ assay. The error bars indicate the s.d. from three independent experiments. *, *P* < 0.05; ***, *P* < 0.001. (E) IFITM3-knockdown cells were infected with the same amount of rVSV-ΔG-EGFP-G for 12 h. Viral transduction was assessed via fluorescence microscopy. (F) IFITM3-knockdown cells were infected with equal amounts of rVSV-ΔG-EGFP-MERS-CoV-S or rVSV-ΔG-EGFP-SARS-CoV-2-S. Samples were analyzed via fluorescence microscopy or flow cytometry. (G) Huh7.5 cells were treated with various doses of IFN-β for 12 h and then infected with the same amount of rVSV-ΔG-EGFP-PEDV-S. At 12 hpi, the samples were analyzed via flow cytometry.

**Figure S5. Sequence alignment of the human and porcine IFITM1, IFITM2 and IFITM3 proteins**. Conserved amino acids among the three human and porcine IFITMs are shaded in blue. The transmembrane (TM) domains are highlighted with red dashed boxes. The conserved CD225 domain is underlined with a green line. Residues for S-palmitoylation and ubiquitination are indicated by red triangles and red triangles, respectively.

**Figure S6. Interaction of the PEDV S1 protein with human IFITM3 and porcine IFITM1.**Coimmunoprecipitation (co-IP) assays were performed to assess the interaction between the PEDV S1 protein and human IFITM3 or porcine IFITM1 proteins. HEK293T cells were cotransfected with plasmids encoding PEDV S1-Fc or PEDV S2-Fc and HA-tagged IFITM proteins. At 24 h posttransfection, the cells were harvested. Immunoprecipitation was conducted using an Fc tag antibody, and the presence of IFITM proteins was detected by Western blotting (IB: Anti-HA). Whole-cell lysates (WCLs) were analyzed to confirm expression levels (IB: Anti-Fc, Anti-HA, Anti-β-Tubulin).

**Figure S7. IFITM membrane topology models illustrating different proposed locations of the N-terminal domain (NTD), C-terminal domain (CTD), and conserved intracellular loop (CIL).** (A) Model 1 depicts both NTD and CTD extracellular, connected by two transmembrane domains (TM1, TM2) and the CIL. (B) Model 2 shows NTD, CTD, and CIL all located in the cytoplasm, with TM1 and TM2 not spanning the membrane. (C) Model 3 positions the NTD and CIL intracellularly, with the CTD extracellular, suggesting TM1 does not span the membrane while TM2 does.
