# Supplementary material for: IFITM proteins are key entry factors for porcine epidemic diarrhea coronavirus
Source: J Virol. 2025 May 12;99(6):e02028-24. doi: 10.1128/jvi.02028-24 (PMC12172462; doi:10.1128/jvi.02028-24)
Supplement: Table S1 — Oligo sequences used in this study. [file jvi.02028-24-s0009.docx]

Table S1. Oligo sequences used in this study

| Name^a^ | Sequence (5’-3’)^b^ | Usage |
| --- | --- | --- |
| sgRNA-IFITM3-Human-1 | ATCCCGATTTGACAAATGCC | Knockout |
| sgRNA-IFITM3-Human-2 | GGGGGCTGGCCACTGTTGAC |  |
| shRNA-Scramble | CCTAAGGTTAAGTCGCCCTCG | Knockdown |
| shRNA-IFITM3-Human-1 | CCAACTATGAGATGCTCAAGG |  |
| shRNA-IFITM3-Human-2 | CCTCATGACCATTCTGCTCAT |  |
| HA-IFITM1-Human-F | ttcaggtgtcgtgaggatccATG*TACCCATACGACGTCCCAGACTACGCT*CACAAGGAGGAACATGAG | PCR |
| HA-IFITM1-Human-R | cggccgccctcgaggaattcCTAGTAACCCCGTTTTTCCT |  |
| HA-IFITM2-Human-F | ttcaggtgtcgtgaggatccATG*TACCCATACGACGTCCCAGACTACGCT*AACCACATTGTGCAAACC | PCR |
| HA-IFITM2-Human-R | cggccgccctcgaggaattcCTATCGCTGGGCCTGGAC |  |
| HA-IFITM3-Human-F | ttcaggtgtcgtgaggatccATG*TACCCATACGACGTCCCAGACTACGCT*AATCACACTGTCCAAACC | PCR |
| HA-IFITM3-Human-R | cggccgccctcgaggaattcCTATCCATAGGCCTGGAA |  |
| HA-IFITM1-Porcine-F | ttcaggtgtcgtgaggatccATG*TACCCATACGACGTCCCAGACTACGCT*ATCAAGAGCCAGCACG | PCR |
| HA-IFITM1-Porcine-R | cggccgccctcgaggaattcctagtagcctctgttactct |  |
| HA-IFITM2/3-Porcine-F | ttcaggtgtcgtgaggatccATG*TACCCATACGACGTCCCAGACTACGCT*AACTGCGCTTCCCAGC | PCR |
| HA-IFITM2/3-Porcine-R | cggccgccctcgaggaattcCTAGTAGCCTCTGTAATCCTTTATG |  |
| IFITM3-Porcine-TC-F | ttcaggtgtcgtgaggatccATGAACTGCGCTTCCCAG | PCR |
| IFITM3-Porcine-TC3-R | cggccgccctcgaggaattcCTAGTAATCCTTTATGAGCTGCAGAAC |  |
| IFITM3-Porcine-TC6-R | cggccgccctcgaggaattcCTATATGAGCTGCAGAACTGCTT |  |
| IFITM3-Porcine-TC9-R | cggccgccctcgaggaattcCTACAGAACTGCTTGGAAAATTACCAG |  |
| IFITM3-Porcine-TC12-R | cggccgccctcgaggaattcCTATTGGAAAATTACCAGGGAGCC |  |
| IFITM3-Porcine-TC13-R | cggccgccctcgaggaattcCTAGAAAATTACCAGGGAGCCAGT |  |
| IFITM3-Porcine-TC14-R | cggccgccctcgaggaattcCTAAATTACCAGGGAGCCAGT |  |
| IFITM3-Porcine-TC15-R | cggccgccctcgaggaattcCTATACCAGGGAGCCAGTGG |  |
| IFITM3-Porcine-TC16-R | cggccgccctcgaggaattcCTACAGGGAGCCAGTGGTG |  |
| IFITM3-Porcine-TC17-R | cggccgccctcgaggaattcCTAGGAGCCAGTGGTGCAAAC |  |
| IFITM3-Porcine-TC18-R | cggccgccctcgaggaattcCTAGCCAGTGGTGCAAACGA |  |
| PEDV-N-qPCR-F | GAAGGCGCAAAGACTGAACC | Virus RNA copies PCR |
| PEDV-N-qPCR-R | TTGCCATTGCCACGACTCCT |  |

Note: ^a^ F denotes forward PCR primer; R denotes reverse PCR primer.

^b^ homologous arms are lowercase and HA tags are in italics.
